# Production, acceptability, and online comprehension of Spanish differential object marking by heritage speakers and L2 learners

**DOI:** 10.3389/fpsyg.2023.1106613

**Published:** 2023-04-20

**Authors:** Begoña Arechabaleta Regulez, Silvina Montrul

**Affiliations:** ^1^Department of Romance Languages and Literatures, University of Chicago, Chicago, IL, United States; ^2^Department of Spanish and Portuguese, University of Illinois at Urbana-Champaign, Urbana, IL, United States

**Keywords:** differential object marking, Spanish, variation, L2 speaker, heritage speaker, production, acceptability, processing

## Abstract

We analyzed the production, acceptability and online comprehension of Spanish differential object marking (DOM) by two groups of bilingual speakers living in the U.S.: heritage speakers and L2 learners. DOM is the overt marking of direct objects that are higher on the animacy and referentiality scales, such as animate and specific objects in Spanish, marked by the preposition *a* (*Juan ve a María* ‘Juan sees DOM María’). Previous studies have reported variability and high omission rates of obligatory DOM in bilingual situations where Spanish is in contact with a non-DOM language.Our study combined different methodologies to tap knowledge of DOM in the two groups. The results showed that heritage speakers and L2 learners (1) exhibited variability with DOM in production (in two oral tasks), comprehension (in an acceptability judgement task), and processing (in an eye-tracking reading task); (2) can integrate DOM into their production, judgments and processing, but they do so inconsistently, and (3) type of task and type of sentence each have an effect on speakers’ use of DOM.

## Introduction

Inflectional morphology is an area of significant variability in some bilingual grammars. It is still not known whether this variability is due to problems at the level of linguistic representations in the weaker, or non-dominant language or whether it is access to linguistic representations for comprehension, production and processing that is at the root of such variability. Both second language (L2) learners of Spanish and heritage speakers of Spanish have been shown to have difficulty with differential object marking (DOM), the overt morphological marking of animate, specific direct objects with the preposition “a” (Farley and McCollam, 2004; McCollam Wiebe, 2004; Montrul, 2004, 2010; Guijarro-Fuentes and Marinis, 2007; Bowles and Montrul, 2008, 2009; Guijarro-Fuentes, 2012; Montrul and Sánchez-Walker, 2013; Arechabaleta-Regulez, 2014). These studies have found high rates of omission of DOM in bilingual situations, where Spanish is in contact with a non-DOM language. In such situations, speakers omit DOM with animate specific objects, as in *Caperucita Roja visitó la abuelita* ‘Little Red Riding Hood visited ø her grandmother’ (Montrul and Sánchez-Walker, 2013). Such omission of DOM in obligatory contexts has been reported in U.S. Spanish in contact with English (Montrul, 2004; Montrul and Sánchez-Walker, 2013) and in Peru Spanish in contact with Quechua (Sánchez, 2003). Although it is possible that DOM omission in these cases may be related to the fact that the other language does not exhibit DOM, DOM omission has also been reported in some monolingual contexts, as in Dominican (Lunn, 2002; Bullock and Toribio, 2004) and Cuban Spanish (Alfaraz, 2011). So, the nature of this variability is still begging for an explanation.

### Spanish differential object marking

Spanish is similar to many other languages including Romanian, Hindi or Turkish in that overt case-marking happens to mark differentially some but not all objects by prepositions or postpositions. This phenomenon is known as Differential Object Marking (DOM). The object that is marked is semantically prominent and is distinguished from subjects by overt marking (Aissen, 2003). In Spanish, animate and specific (definite) objects are marked with DOM. For example, sentence (1) shows that because the direct object is [+animate] and [+specific] (definite)^1^, DOM is required. When the direct object is [+animate] and [− specific], DOM is not required (2) and DOM can either be used or omitted. However, when the direct object is [−animate] and [+specific] (3) or [−animate] and [−specific] (4) DOM is not used.

(1) *Mario vio a la doctora* ‘Mario saw the- DOM doctor [+animate] and [+specific]

(2) *Mario vio (a) una doctora* ‘Mario saw a (DOM) doctor [+animate] and [−specific]

(3) *Mario vio el carro* ‘Mario saw the car’ [−animate] and [+specific]

(4) *Mario vio un carro* ‘Mario saw a car’ [−animate] and [−specific]

Even though animate objects are typically marked and inanimate objects are not, there exists some variation in the use of DOM in both monolingual and bilingual contexts. For example, several Spanish varieties in Latin America appear to show a slight tendency to overextend DOM to inanimate objects. A sentence like (5) in Rioplatense Spanish or (6) in Mexican Spanish are acceptable for some speakers in those varieties, while the same sentences are ungrammatical in other varieties, such as Peninsular Spanish. Moreover, in other Spanish varieties, the opposite development has been observed: DOM retraction. DOM retraction refers to the omission of DOM in contexts where DOM is usually used. The omission of DOM with animate and specific (definite) objects has been observed in some monolingual contexts (Lunn, 2002) as well as in some bilingual contexts (Montrul and Sánchez-Walker, 2013). This study focusses on this DOM omission by bilingual speakers (heritage speakers and L2 learners) living in the US.

(5) *Chocó al coche* (Sánchez and Zdrojewski, 2013 Rioplatense Spanish)

‘He hit the car’.

(6) *Cosecharon al maíz* (von Heusinger and Kaiser, 2005 Mexican Spanish)

‘They harvested-DOM the corn’.

### Heritage speakers and L2 learners of Spanish

Most Spanish language classrooms in the U.S. consist of both English-speaking students learning Spanish as an L2 (L2 learners of Spanish) as well as students who were raised hearing Spanish spoken at home (heritage speakers of Spanish). Heritage speakers are typically simultaneous and early successive bilinguals who are exposed to a minority language at home since birth and to a majority language in the community since birth or in childhood (Valdés, 2001; Montrul, 2004, 2016). As adults, heritage speakers tend to be dominant in the majority language and weaker in their heritage language, as assessed by both self-reports (Montrul, 2022), independent measure of proficiency (Montrul, 2016), and linguistic tasks (Montrul and Ionin, 2010). L2 learners, on the other hand, are usually sequential bilinguals who grow up exposed to the majority language and only begin acquiring an L2 during or after puberty. L2 learners and heritage speakers’ experience with the weaker language is different. Table 1 summarizes the main features of the two types of acquisition (heritage language and L2) from which differences and similarities between heritage speakers and L2 learners can be drawn. While heritage speakers are exposed to Spanish during childhood, typically through an aural medium and in a naturalistic context (home), L2 learners are exposed to Spanish during or after puberty in a formal context (classroom) with a strong emphasis on reading and writing activities as well as structured grammar explanations, activities and feedback. Therefore, L2 learners, but not heritage speakers, tend to be very literate in their L2 and have highly developed metalinguistic knowledge of the target language. Metalinguistic knowledge is typically defined as the explicit and declarative knowledge the speakers have about the language. Heritage speakers, on the other hand, usually have less developed literacy skills and less metalinguistic knowledge of their heritage language than their majority language. Motivation to learn and maintain the language is another important difference between these two types of speakers. The main motivations for heritage speakers to regain their language are to maintain their heritage language, strengthen family connections, and reinforce their identity (Reynolds et al., 2009). In contrast, L2 learners usually seek to improve their grammatical skills (Mikulski, 2006) and be able to communicate with people who can speak the target language (Reynolds et al., 2009). They also seek professional opportunities (Beaudrie and Ducar, 2005; Alarcon, 2010; Carreira and Kagan, 2011). Finally, heritage speakers may not want to use their heritage language due to the social stigma attached to their Spanish which debilitates their view of themselves as Spanish speakers (Kutlu and Kircher, 2021).

**Table 1:** Characteristics of heritage language and L2 acquisition.

Time	Early exposure	Late exposure (during or after puberty)
Setting	Naturalistic (home)	Instructed (classroom)/ study-abroad
Mode	Aural Input	Aural and Written Input
Errors	Developmental and transfer errors	Developmental and transfer errors
Fossilization	Typical	Typical
Motivation	Yes	Yes
Outcome	Variable	Variable

Despite these differences in language experience, heritage speakers and L2 learners also share many similarities. For example, when using the target language, both types of speakers tend to show morphological variability due to the influence of the majority language. Previous research comparing heritage speakers and L2 learners has suggested that age of acquisition alone cannot explain the main differences between the two groups (Au et al., 2002; Benmamoun et al., 2010). According to the notion “earlier is better,” heritage speakers should always outperform L2 learners because they are exposed to the language at an earlier age. However, this is simply not the case (Au et al., 2002; Montrul et al., 2008), because language experience shapes their knowledge as well and this is manifested in different tasks and the modality in which the language is tested. While heritage speakers usually outperform L2 learners in oral tasks of morphosyntax, results vary and often depend on the type of task. Heritage speakers tend to have an advantage with tasks that tap languge implicitly and minimize metalinguistic knowledge (Bowles, 2011); L2 learners, by contrast have an advantage with tasks that focus on explicit knowledge of the language and are more metalinguistic. The fact that heritage speakers and L2 learners perform differently depending on the degree of explicitness or implicitness of the task suggests that performance is heavily influenced by language experience (Bowles, 2011). That is why in order to understand the nature of their linguistic knowledge, it is important to use tasks that tap into participants’ explicit and implicit knowledge.

It has been common to test implicit knowledge *via* oral tasks because language production unfolds over time. However, analyzing participants’ free production in oral tasks is often insufficient to measure implicit knowledge accurately. For instance, participants still have opportunities to resort to their explicit knowledge in oral tasks, especially when the task is untimed and participants can monitor and repair their performance (Jiang, 2004).

The use of online processing techniques are essential to offer evidence of implicit knowledge. Unlike offline tasks, online tasks tap into individuals’ implicit knowledge by analyzing the actual processing mechanisms that are being used during comprehension or production in real time (Field, 2004). Thus, these online tasks measure implicit real-time behavior/reactions as opposed to measuring potential ‘learned’ knowledge of heritage language or the L2. Properly examining access to implicit knowledge is important because, according to certain language processing theories, implicit knowledge can only be accessed if one is exposed to the language early in life [e.g., The Declarative/Procedural Model (Ullman, 2004); The Shallow Structure Hypothesis (Clahsen and Felser, 2006)]. Moreover, access to implicit knowledge is thought to be central to acquiring native-like competence in both L1 and L2 acquisition (Krashen, 1982). If heritage speakers do not show the same advantages over L2 learners when tested with online processing tasks as they do when tested with oral tasks, this would suggest that early exposure in a naturalistic context is not enough to achieve a high level of implicit knowledge in that language. If this is the case, limited use and exposure to the language in late childhood and adolescence may be affecting their competence.

### Omission of DOM by L2 learners and heritage speakers

Previous studies have consistently shown that both heritage speakers and L2 learners omit the *a*-marker with animate objects (Farley and McCollam, 2004; McCollam Wiebe, 2004; Montrul, 2004; Guijarro-Fuentes and Marinis, 2007; Bowles and Montrul, 2008, 2009; Montrul, 2010; Guijarro-Fuentes, 2012; Montrul and Sánchez-Walker, 2013; Arechabaleta-Regulez, 2014). For example, Montrul (2010) compared heritage speakers and L2 learners on the acquisition of DOM. Montrul investigated whether age of onset of acquisition and/or influence from their dominant language, English, was preventing heritage speakers and L2 learners from fully acquiring DOM. Heritage speakers (*n* = 67) and L2 learners (*n* = 72) were divided into three groups depending on their Spanish proficiency: advanced (Heritage Speakers = 32, L2 = 25), intermediate (Heritage Speakers = 26, L2 = 25) and low (HS = 13, L2 = 22). Heritage speakers and L2 learners were compared to a group of monolingually-raised native speakers from different Spanish-speaking countries. Participants completed two main tasks: an oral narrative task (Montrul, 2004) and an acceptability judgment task. Results for the oral narrative task showed that heritage speakers and L2 learners at all proficiency levels omitted DOM with animate objects, while the native speakers did not. However, the L2 learners produced almost twice the amount of omissions (46.9%) as the heritage speakers (26.5%). Moreover, results also showed that advanced heritage speakers did not differ significantly from the native speaker control group, which suggests that proficiency is an important factor when comparing heritage speakers to monolingually-raised native speakers. As for the AJT, results showed that, overall, heritage speakers and L2 learners accepted sentences with DOM omission and animate objects, but the control group did not. In this task, L2 learners behaved more like the native speakers, as heritage speakers, regardless of proficiency, accepted sentences with DOM omission and animate objects significantly more often. Therefore, the two groups differed significantly from the native speakers, but the L2 learners outperformed the heritage speakers, especially at lowest levels of proficiency. Montrul (2010) concluded from the results of the two tasks that DOM is subject to incomplete acquisition or attrition for both heritage speakers (Montrul and Bowles, 2009, 2010) and L2 learners (McCollam Wiebe, 2004; Bowles and Montrul, 2009). Montrul also noted the importance of using different tasks when comparing heritage speakers and L2 learners. In the oral task, the heritage speakers showed an advantage over the L2 learners, but in the written task, the L2 learners showed an advantage over the heritage speakers. Finally, Montrul suggested that DOM omission can easily be attributed to transfer from English. Spanish, unlike English, is a language with rich inflection, and rich agreement co-occurs with the possibility of non-canonical word order. In those cases, Spanish relies on case marking to indicate thematic roles. Thus, DOM is crucial to understand *who* is doing *what*, especially when the object is animate. English word order, on the other hand, is relatively fixed. Thus, word order usually conditions thematic interpretations in English.

In fact, the omission of case marking is heavily influenced by the word order flexibility of the language. To test the correlation between word order and case marking in a language, Fedzechkina et al. (2015) exposed learners to two miniature artificial languages. Both languages contained case marking, but while one language had flexible word order, the other had fixed word order. Results showed that learners who were exposed to the language with flexible word order used case marking more often than the learners who were exposed to the language with fixed word order. The learners made changes to the artificial languages that are compatible with language universals; that is, grammatical patterns that are prone to happen crosslinguistically. In cases where speakers have grammatical cues that are highly informative (e.g., word order), other cues become redundant and are thus omitted (e.g., case marking). Indeed, Lunn (2002) has suggested that DOM is disappearing from Dominican Spanish because of another innovation occurring in this dialect: Dominican native speakers appear to use a stricter SVO word order, and thus direct objects are expected to appear after the verb. Therefore, using DOM to disambiguate thematic roles is becoming uninformative. The tradeoff between word order and case marking as a cue to thematic roles is also discussed in the Unified Competition Model (UCM; MacWhinney, 2005; see also the Competition Model of Bates and MacWhinney, 1987).

The omission of DOM with animate objects that has been observed in both heritage speakers and L2 learners is compatible with these language universals. Perhaps, DOM retraction may be a consequence of a change in the word order possibilities of Spanish in contact with English. In other words, the Spanish of the United States may be acquiring a more fixed SVO word order similar to Dominican Spanish. Still, a major question remains: Is DOM disappearing across the board in these varieties or only in contexts where case marking may be less informative (sentences with canonical word order)?

The aim of this study is to investigate whether heritage speakers and L2 learners, who often omit DOM in production and grammaticality judgments, do not process DOM during sentence processing. Unlike previous studies that have mostly focused on SVO sentences, the present study examines whether omission of DOM occurs with canonical and/or non-canonical word order sentences. The majority of studies on heritage speakers and L2 learners have not examined the interaction between word order and DOM. However, heritage speakers and L2 learners may show omission of DOM only in contexts where case marking is less informative, as in SVO sentences. If tested in contexts where DOM is critical for comprehension (sentences with non-canonical word order), heritage speakers and L2 learners may not show the same extent of DOM omission. Previous research on the processing of DOM by heritage speakers and L2 learners of Spanish suggests that DOM omission is reflected in speakers’ processing mechanisms. When exposed to ungrammatical sentences with unmarked animate objects, neither heritage speakers nor L2 learners show any sensitivity to ungrammaticality (Jegerski, 2015, 2018).

Arechabaleta-Regulez (2016), investigated heritage speakers’ processing of DOM in sentences with canonical (SVO) and non-canonical (VSO) word order. Results of an eye-tracking during reading task demonstrated that heritage speakers were more sensitive to DOM omission with non-canonical VSO word order than with canonical SVO sentences. This suggests that heritage speakers rely on word order and ignore case marking with canonical word order sentences, possibly due to transfer from their dominant language (English). However, with non-canonical word order sentences, heritage speakers appeared to utilize DOM as an informative cue to word order. Therefore, omission of DOM was evident in their processing of canonical word order sentences but not in their processing of non-canonical word order sentences. Building on Arechabaleta-Regulez (2016), this study examines whether L2 learners behave like heritage speakers in their processing of DOM. We predicted that their different language learning experiences regarding timing (before vs. after the critical period) and context of acquisition (naturalistic vs. formal environment), may affect their processing. However, we also test production and judgments of DOM because it is well known that heritage speakers and L2 learners tend to show DOM omission (e.g., Montrul and Sánchez-Walker, 2013). In this study, the tasks provide comprehensive information related to participants’ production, acceptance and online comprehension of DOM. The importance of analyzing bilinguals’ productive and receptive knowledge is to understand potential dissociations and asymmetry between speakers’ production, acceptability and processing.

Participants completed the reading task with eye-tracking first, followed by the oral tasks, first the narrative task and then the elicitation task, and finally the AJT. After completing these tasks, participants also completed the background questionnaire and a written Spanish Proficiency test. Proficiency scores were included as covariates to assess the extent to which proficiency affected participants’ production, acceptability and online comprehension of DOM. The following sections describe each task in greater detail, including the corresponding research questions, hypotheses and results. Rather than following the exact order in which participants completed the tasks, the discussions are arranged so that the most innovative findings are discussed last.

## Methodology

### Participants

Thirty-five heritage speakers and 42 L2 learners were recruited. All participants were between the ages of 18 and 25 (average age 21.3). In order to participate in the study, heritage speakers were required to: (1) have been born in the U.S. (they were all second generation); (2) have been exposed primarily to Spanish in early childhood or to both Spanish and English and (3) be of Mexican origin to the greatest extent possible (either one parent or both were from Mexico). L2 learners were required to: (1) have been born in the U.S.; (2) have been exposed to Spanish in a formal context but not earlier than the age of 10 (L2 speakers reported that they had been exposed to various Spanish dialects depending on their teachers) and (3) not speak any other second language besides Spanish. We were primarily interested in testing heritage speakers and L2 learners with an intermediate to high proficiency in Spanish. Heritage speakers and L2 leaners completed a background questionnaire to determine whether they met all of these requirements and an adapted version of the DELE (Diploma of Spanish as a Foreign Language) proficiency test as an independent measure of proficiency in Spanish (see Table 2).

**Table 2:** Background questionnaire information.

Participants	*N*	Age	AoA of Spanish	AoA of English	DELE scores
Heritage Speakers	35	19.3 (18–22)	Birth	2.2(0–4)	39.76 (21–46)
L2 Learners	42	20.2(18–24)	12.2 (10–14)	Birth	26.62 (13–46)

**Figure 1:**
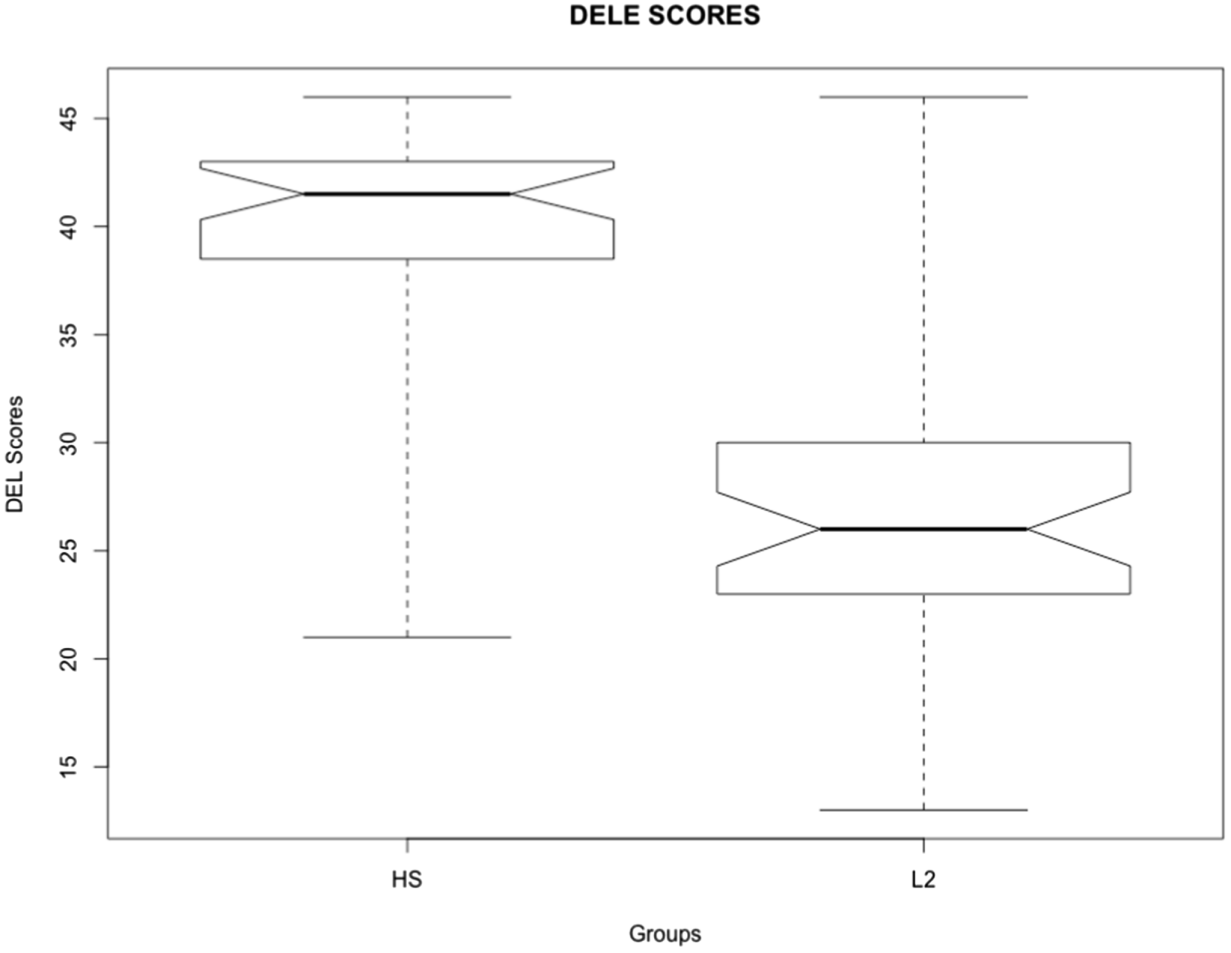
DELE scores for the heritage speakers and L2 learners.

When comparing the results obtained in the DELE test, there was a significant effect (*β* = −12.66, SE = 1.46, *p* < 0.0001) as heritage speakers scored significantly higher than the L2 learners. Moreover, as Figure 1 shows, the dispersion of the scores varied. While most of the heritage speakers scored above 35 points, most of the L2 learners scored between 20 and 30 points out of a maximum of 50 points. Before testing the participants’ language processing, it is also important to test their production and judgments of DOM. Therefore, participants completed two oral tasks and an acceptability judgment task (AJT). No study has used all these methodologies to examine oral production, judgment and sentence processing during reading in the two groups. Analyzing bilinguals’ productive and receptive knowledge is critical to understand potential dissociations and asymmetries between their production, acceptability and processing.

### Procedure

Participants arrived at the laboratory where they first read and signed a consent form. Then, they began the study by completing the reading task with eye-tracking, for which a portable eye-tracker (Eye Link SR Research, Ltd.; Ottawa, Canada) with remote desktop camera sampling at 500 Hz was used. The eye-tracker was used in a diagnostic manner because it recorded and analyzed participants’ eye position while reading sentences. Subjects were seated 50 cm from the monitor with their chin/head rest. It is important to use a chin/head rest to increase accuracy of measurement (Carter and Luke, 2020). Sentences were presented in 18-point Courier font, left-aligned on the display. Before the task began, a calibration procedure was carried out to accurately track participants’ eye-movements. During this initial process, participants were instructed to fix their gaze on a set of nine fixation points (black dots) displayed on the screen at known locations. While they were doing this, the positions of their eyes were recorded. If there were no errors when the calibration was performed, the computer then “validated” the information before subjects could begin the actual test. A calibration was accepted if average error was less than 1 degree of visual angle and calibration was as necessary during the experiment.

Next, participants completed a practice session, which consisted of 8 trials, following the same procedure as the actual study to familiarize participants with the eye-tracker and the response controller. The structure of each trial was as follows: first, a white screen with a black dot, the central fixation point, appeared in the left middle of the screen. Participants were told to look at this point immediately prior to pressing a button on a controller, which prompted a sentence to appear on the screen. After reading the sentence, participants pressed the button again to continue to a comprehension question related to the sentence they had previously seen. Participants used one of two buttons to respond ‘yes’ or ‘no’ to the comprehension questions after each trial. After the practice session, participants were instructed to move their head as little as possible during the experiment to ensure accurate tracking of their eye movements. Participants were also informed that they would be allowed to take three breaks during the experiment. If participants decided to take a break, and thus, moved their chin, recalibration was performed again. The eye-tracker machine recorded all movements of each participant’s right eye between the appearance of the white screen with the black point, indicating the beginning of a new trial, and the disappearance of the sentence, when a participant pressed the button to proceed to the comprehension question. In total, this task lasted between 30 and 45 min.

After the reading task with eye-tracking, participants completed the oral task in two parts: first the narrative task, then the elicitation task. Participants were seated in front of a laptop computer and their answers were recorded by the same laptop for both portions. For the narrative task, participants were asked to narrate the story in Spanish based on the pictures with as many details as possible. They advanced through the presentation at their own pace while their narration was continuously recorded. This task did not take longer than 10 min. The participants then completed the elicited production task, which took less than 10 min.

After the two oral tasks, participants completed the acceptability judgment task (AJT) using the same laptop they used for the oral tasks. Before starting the AJT, participants were told to read the sentences as carefully and as quickly as possible and to rely on their first instinct. The sentences were presented visually, and participants had as much time as they wanted to read and judge the sentences. They were instructed to rate the sentences on a scale of 1 to 5 by pressing a button on the computer, with 1 indicating completely unacceptable and 5 totally acceptable. A rating of 3 represented ‘undecided’. Participants completed the task within 30 to 40 min. Finally, participants completed the background questionnaire, which took about 20 to 30 min. In total, it took participants between 1.5 to 2 h to complete all of the tasks. Thus, all participants completed the most implicit tasks first (i.e., the reading task with eye-tracking) and the most explicit tasks last (i.e., the AJT).

The following sections describe each task in greater detail, including the corresponding research questions, hypotheses and results. Rather than following the exact order in which participants completed the tasks, the discussions are arranged so that the most innovative findings are discussed last.

### Oral tasks: Narrative task and elicited production task

First, we asked to what extent heritage speakers and L2 learners omit DOM in obligatory contexts in oral production, and whether their performance depended on the implicit or metalinguistic nature of the task, as found in previous studies. Two oral tasks—an oral narrative task and an elicited production task—measured participants’ oral production of Spanish DOM. For the narrative task, participants narrated the children’s story ‘Little Red Riding Hood’ (from Montrul, 2004). Participants were provided with 14 colorful pictures of the story *via* a PowerPoint slideshow and were asked to narrate the story using the preterit tense while providing as much detail as possible based on the pictures. The pictures contained many animate and inanimate referents as objects. Because participants are usually more concerned with *what* to say (meaning of the story) rather than *how* to say it (grammar) when completing narrative tasks, this task provides semi-spontaneous data, perhaps comparable to what one can elicit with sociolinguistic interviews.

**Figure 2:**
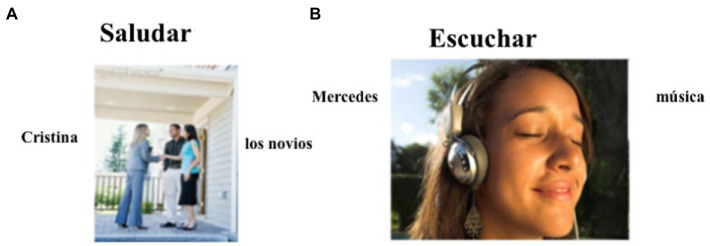
Sample of items used in the oral elicitation task: **(A)** shows the picture used for the verb *saludar* ‘to greet’; **(B)** shows the picture used for the verb *escuchar* ‘to listen’ [reproduced with permission from Arechabaleta-Regulez and Montrul (2021)].

In the elicitation task, participants were presented with a picture with a verb and animate and inanimate NPs as subjects and objects on a computer screen and were asked to produce a sentence describing the picture using the verb and NPs given (see Figure 2). Participants were told to conjugate the verb in the preterite tense, so the presence or absence of DOM could be perceived. In total, participants were presented with 24 pictures: 12 with animate objects and 12 with inanimate objects. Another 12 pictures were included as fillers. The fillers prompted participants to use different constructions (e.g., sentences with *gustar*-type verbs). We believe that in this task participants have less freedom to produce spontaneous speech as they are given some of the words they need to use.

Both heritage speakers and L2 learners were expected to show a significant rate of DOM omission in their production. However, following previous research on the production of DOM, overall, heritage speakers were expected to show less DOM omission than the L2 learners; especially because proficiency an important factor (Montrul, 2010). Participants with a higher proficiency were expected to show fewer ungrammatical unmarked animate objects. Overall, participants were not expected to extend DOM to inanimate objects. With respect to task effects, the L2 learners were expected to show more DOM omission in the narrative task than in the elicitation task. The elicitation task is more explicit, and thus participants may rely more on their explicit knowledge and use their metalinguistic knowledge while completing this task. As for the heritage speakers, they were expected to show the opposite pattern; namely, more omission of DOM in the elicitation task than in the narrative task. While L2 learners seem to perform better in explicit tasks that maximize metalinguistic knowledge, heritage speakers seem to perform better in implicit tasks that minimize metalinguistic knowledge.

## Results: Oral tasks

### Narrative oral task

Participants’ answers were audio recorded and their answers transcribed and coded by a native speaker from Spain. All sentences containing object NPs were analyzed and the objects were coded for animacy and for DOM marking (present or absent). In situations where participants produced unexpected sentences, those sentences were coded as ‘other’ and were removed from the final statistical analyses. An example of a sentence coded as ‘other’ is when participants used the passive voice ‘*El alumno fue castigado’* ‘The student was punished’ instead of the active sentence with DOM *La profesora castigó al alumno’* ‘The teacher punished the student’. Results were analyzed with a bivariate logistic regression with the framework of *glm* (generalized linear model) using R (version 1.1.453 for Mac OS X, R Development Core Team, 2014), with participant and item as random effects and markedness ([+DOM] vs. [-DOM]), animacy of the object (animate vs. inanimate) and group (heritage speakers vs. L2 learners) as fixed effects. All fixed effects were coded as a binary variable using dummy coding (markedness: [+DOM] =1, [-DOM] =0; animacy of the object: animate object = 1, inanimate object = 0; group: heritage speakers = 1, L2 learners = 2). Each participant ended up with 4 percentage scores reflecting their use or omission of DOM with either animate or inanimate objects. Proficiency scores were included as covariates to assess the extent to which proficiency affected performance.

Table 3 shows that, as predicted, heritage speakers (8a) and L2 learners (8b) omitted DOM with animate objects; and that heritage speakers showed lower DOM omission rates than the L2 learners.

**Table 3:** Use or omission of DOM with animate and inanimate objects (narrative).

	Heritage Speakers			L2 Learners		
	Total	Marked	Unmarked	Total	Marked	Unmarked
Animate	199 (100%)	160 (80.40%)	39 (19.60%)	222 (100%)	86 (38.74%)	136 (61.26%)
Inanimate	124 (100%)	0 (0%)	124 (100%)	102 (100%)	5 (4.90%)	97 (95.10%)

For heritage speakers, 80.40% of the animate objects were marked, while 19.60% were unmarked. However, for the L2 only 38.74% of the animate objects were marked and 61.26% were unmarked. Unlike native speakers of Mexican Spanish who have been shown to extend DOM to inanimate objects (Arechabaleta-Regulez and Montrul, 2021), these bilingual participants did not show much extension of DOM to inanimate objects. While heritage speakers did not produce any cases of inanimate objects with DOM extension, the L2 learners did so in 5 occasions, as in (7c).

(7)

- [Participant 302] ver su abuela
  see his/her grandmother
  ‘(She) see her grandmother’
- [Participant 254] comio la nina
  ate the girl
  ‘(the wolf) ate the girl
- [Participant 237] mirando a las flores
  staring DOM at the flowers
  ‘(She/he) was staring at the flowers.

**Figure 3:**
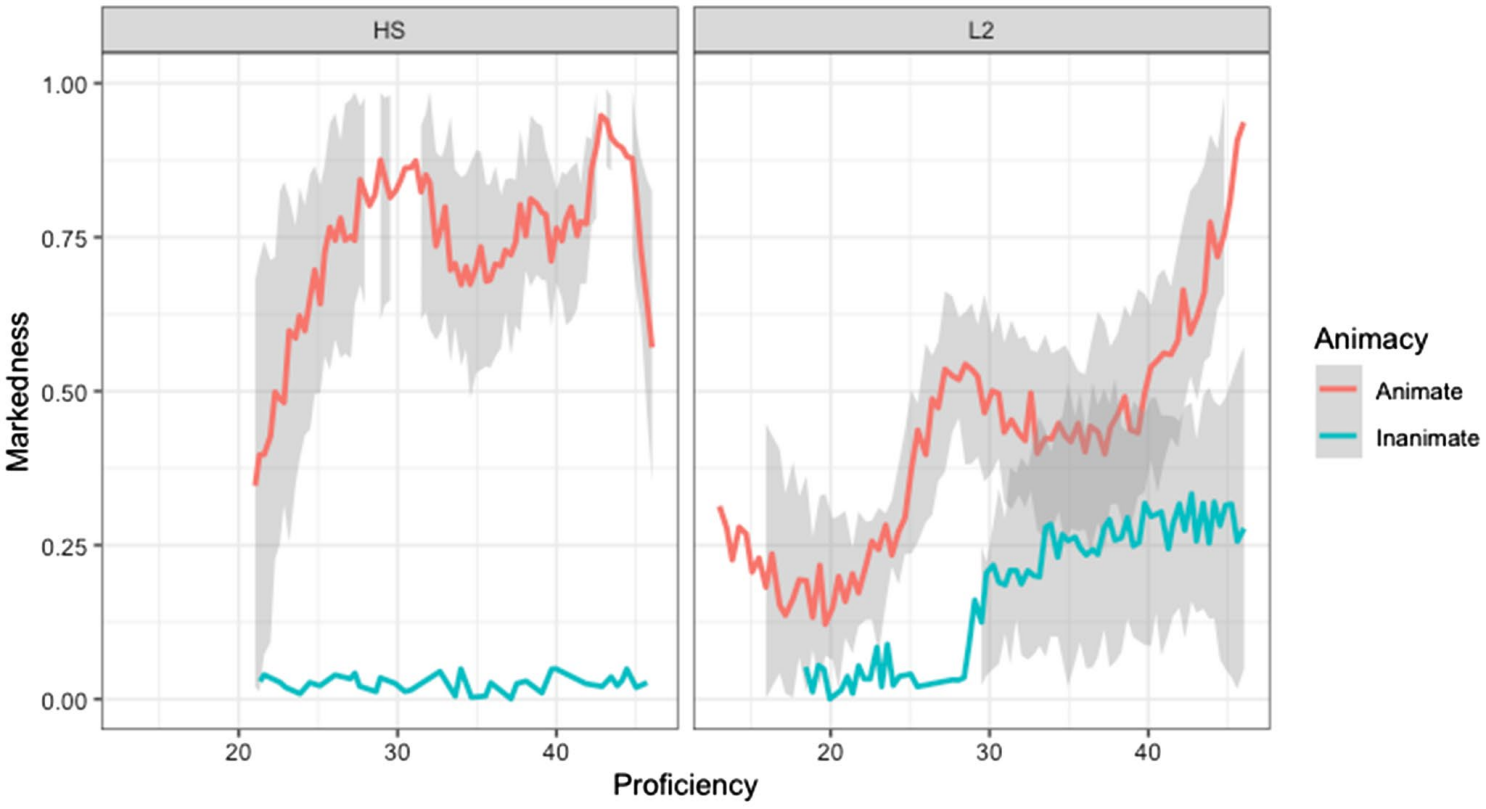
The effect of proficiency on the production of DOM (narrative).

The logistic regression revealed a significant effect of ANIMACY, as animate objects were marked with DOM significantly more than inanimate objects (*β* = −4.24, SE = 0.0008, *p* < 0.0001), and a significant GROUP effect (*β* = −0.72, SE = 0.0008, *p* < 0.0001), as heritage speakers used DOM significantly more often than L2 learners regardless of the animacy of the object. There was also a significant interaction between ANIMACY and GROUP (*β* = 1.96, SE = 0.0008, *p* < 0.0001). Tukey’s multiple comparison test revealed that heritage speakers (*β* = 7.43, SE = 1.11, *p* < 0.0001) and L2 learners (*β* = 3.05, SE = 0.5, *p* < 0.0001) used DOM significantly more often with animate objects than with inanimate objects. However, when comparing the use of DOM with animate objects between the two groups of bilinguals, there was a significant effect (*β* = 1.55, SE = 0.54, *p* = 0.02) as heritage speakers used DOM significantly more often than the L2 learners. As for the use of DOM with inanimate objects, there was not a GROUP effect as the use of DOM was minimal for heritage speakers and L2 learners (*β* = −2.82, SE = 1.22, *p* = 0.09). Finally, there was a significant PROFICIENCY effect (*β* = 0.093, SE = 0.0008, *p* < 0.0001). As Figure 3 shows, participants with higher proficiency used DOM with animate objects more often than participants with lower proficiency.

However, proficiency seems to have a bigger effect on L2 learners than on heritage speakers. Interestingly, for the L2 learners, proficiency also had an effect on the extension of DOM to inanimate objects. It appears that L2 learners with a higher proficiency of Spanish used DOM more with both animate and inanimate objects. Participants may have acquired the rule that states that DOM is used with animate objects, and they are now overextending this rule to inanimate objects. However, heritage speakers did not extend the use of DOM to inanimate objects.

### Oral elicitation task

In total, 31 sentences were coded as ‘other’ and were removed from the statistical analyses. Heritage speakers (8a) and L2 learners (8b) omitted DOM with animate objects; however, heritage speakers again showed less DOM omission than expected: 27.45% of the animate objects were unmarked and 72.55% were marked. L2 learners produced 58.83% of the animate objects unmarked and 61.26% marked. Moreover, there were more cases of DOM extension to inanimate objects in this task by both heritage speakers, as in (8), and L2 learners, as in (5.2d) (see Table 4).

**Table 4:** Use or omission of DOM with animate and inanimate objects (elicitation task).

	Heritage Speakers			L2 Learners		
	Total	Marked	Unmarked	Total	Marked	Unmarked
Animate	412 (100%)	298 (72.33%)	114 (27.66%)	498 (100%)	205 (41.17%)	293 (58.83%)
Inanimate	411 (100%)	46 (11.19%)	365 (88.81%)	496 (100%)	65 (13.08%)	431 (86.92%)

(8)

- [Participant 207] Cristina saludó los novios
  Cristina said hi to the couple
  ‘Cristina said hi to the couple’
- [Participant 322] El ladrón atacó el presidente
  the thief attacked the president
  ‘The thief attacked the president’
- [Participant 311] El viaje llevo al paraguas
  the old mal brought DOM the umbrella
  ‘The old mal brought DOM the umbrella’
- [Participant 213] El hombre besó al trofeo
  the man kissed the DOM trophy
  ‘The man kissed the trophy’.

**Figure 4:**
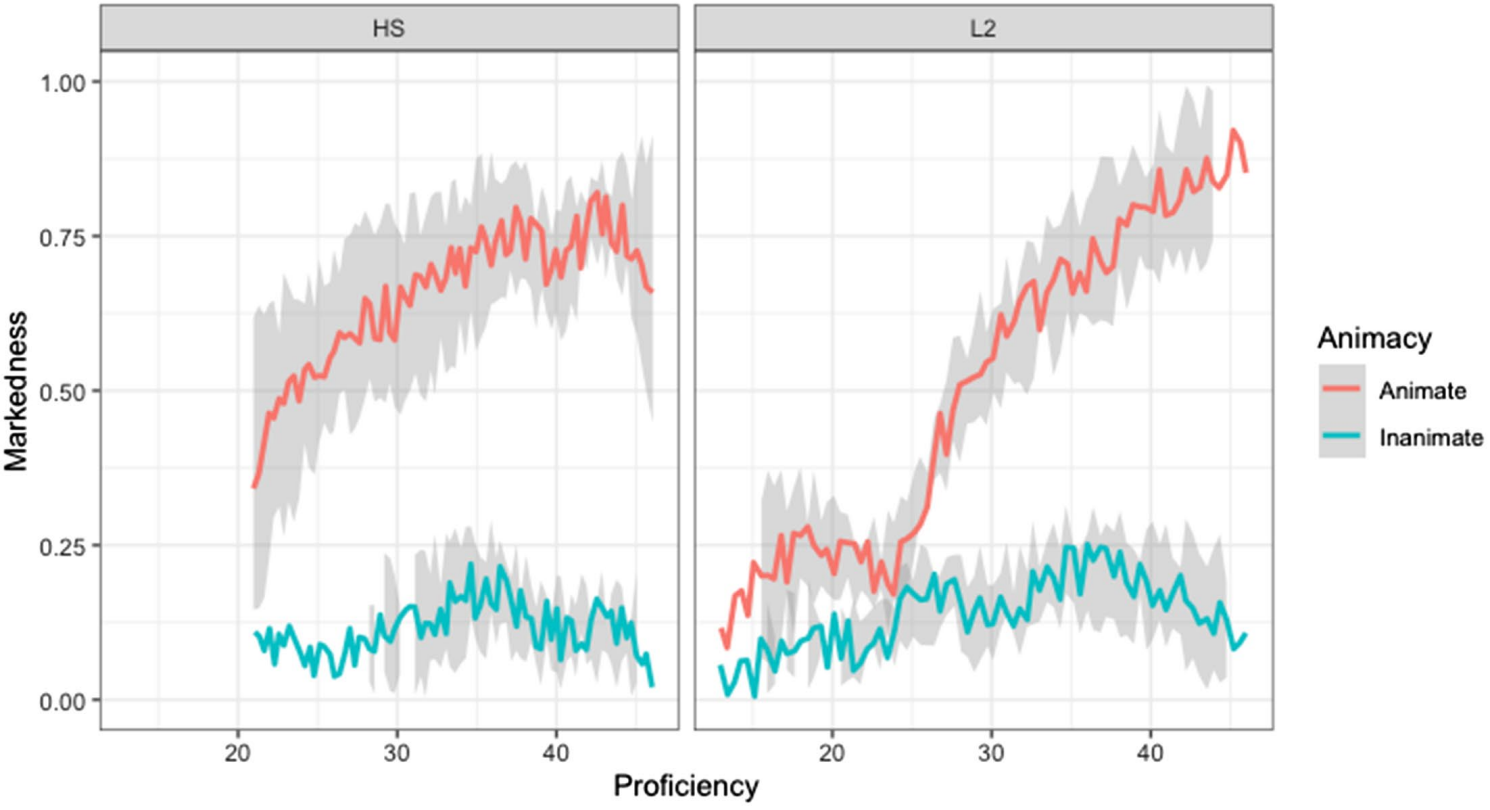
The effect of proficiency on the production of DOM (elicitation task).

The logistic regression revealed a significant effect of ANIMACY (*β* = −2.48, SE = 1.06, *p* = 0.02), because participants marked animate objects significantly more often than inanimate objects overall, and a significant ANIMACY and GROUP interaction (*β* = 1.93, SE = 0.30, *p* < 0.0001). Tukey’s multiple comparison test revealed that heritage speakers (*β* = 4.20, SE = 0.42, *p* = 0.001) and L2 learners (*β* = 2.27, SE = 0.39, *p* < 0.001) used DOM significantly more often with animate objects than with inanimate objects. However, heritage speakers and L2 learners did not significantly differ on either the use of DOM with animate objects (*β* = 0.69, SE = 0.47, *p* = 0.45) or on the use of DOM with inanimate objects (*β* = −1.23, SE = 0.49, *p* = 0.06). That is why in the logistic regression, GROUP did not turn out to be a significant effect (*β* = −0.69, SE = 0.47, *p* = 0.14). Finally, there was a PROFICIENCY effect (*β* = 0.093, SE = 0.0008, *p* < 0.0001), as participants with higher proficiency marked DOM with animate objects more often than participants with lower proficiency. Figure 4 shows that the production of DOM increase as participants’ proficiency increases. In the elicitation task, L2 learners also marked some inanimate objects, but it is not as correlated to proficiency as in the narrative task.

The reason for using two oral tasks was to analyze whether participants’ use of DOM would vary depending on whether they were completing a narrative task or an elicitation task. In order to analyze task effects, results for the animate objects and inanimate objects were analyzed individually with a bivariate logistic regression with the framework of glm in R with participant and item as random effects and markedness ([+DOM] vs. [-DOM]), task (narration vs. elicitation) and group (heritage speakers vs. L2 learners) as fixed effect. Results for the animate objects revealed a significant TASK effect (*β* = 0.65, SE = 0.23, *p* = 0.006) and a significant TASK*GROUP interaction (*β* = −1.06, SE = 0.32, *p* = 0.0009). Tukey’s multiple comparison test revealed a significant difference between the use of DOM by the heritage speakers in the narrative task and in the elicitation task (*β* = −0.65, SE = 0.23, *p* = 0.03), as participants produced DOM with animate objects significantly more in the narrative task than in the elicitation task. However, for the L2 learners there was not a significant effect on the use of DOM between the two tasks. As for the inanimate objects, results revealed a significant TASK effect (*β* = −1.68, SE = 0.60, *p* = 0.005). Tukey’s multiple comparison tests only revealed a significant effect when comparing the use of DOM with inanimate objects in the narrative task and in the elicitation task (*β* = 1.68, SE = 0.60, *p* = 0.02), as heritage speakers used DOM with inanimate objects significantly more often in the elicitation task than in the narrative task. For the L2 learners, there were not any significant comparisons.

#### Summary of results

As hypothesized, participants showed DOM omission in the narrative and in the elicitation task. Nevertheless, the L2 learners produced significantly more unmarked animate objects than the heritage speakers. Moreover, the L2 learners also showed more extension of DOM to inanimate objects than the heritage speakers. Proficiency turned out to be a significant factor, especially for the L2 learners. Participants with a higher proficiency, used DOM significantly more than participants with a lower proficiency in Spanish. Proficiency also had an effect on the extension of DOM to inanimate objects for the L2 learners. L2 learners with a high proficiency in Spanish produced more marked inanimate objects.

### Acceptability judgment task (AJT)

The aim of this task was to test participants’ judgments of grammatical and ungrammatical sentences with DOM in both SVO and VOS sentences. Sentences varied by animacy of the object (animate vs. inanimate) and object marking ([+DOM] vs. [−DOM]) as shown in Table 5.

**Table 5:** Sample sentences used in the AJT.

Direct object	[+DOM]	[−DOM]
Animate	El niño acusó al señor de las gafas azules.	*Diego acogió el estudiante de intercambio.
	‘The kid accused the man with the blue glasses.’	‘Diego welcomed the exchange student.’
Inanimate	El joven apreció al esfuerzo económico por parte de sus padres.	La actriz dibujó el carro de sus sueños
	‘The young boy appreciated the economic effort that his parents made.’	‘The actress drew her dream car.’

Based on previous studies (Guijarro-Fuentes and Marinis, 2007; Montrul and Sánchez-Walker, 2013), we predicted that the bilingual speakers would accept sentences with animate objects and DOM (*El niño acusó al señor de las gafas azules*) as well as sentences with unmarked inanimate objects (*La actriz dibujó el carro de sus sueños*), and would show more variability rejecting ungrammatical sentences with animate objects and DOM omission (*Diego acogió el estudiante de intercambio).

As in previous studies (Montrul, 2010; Guijarro-Fuentes, 2012), proficiency was expected to play a role on L2 participants’ rating as participants with a higher proficiency in Spanish were expected to show less acceptance of DOM omission. Finally, word order was also expected to play a role. Higher rejection of DOM omission with animate objects was expected in sentences with non-canonical word order, as DOM is more informative. With sentences with inanimate objects, participants were expected to reject ungrammatical sentences with DOM and to accept unmarked objects, which are grammatical (Jegerski, 2018). Finally, as this is a metalinguistic task, L2 learners were expected to reject ungrammatical DOM omission with animate objects and ungrammatical DOM extension to inanimate objects more than heritage speakers overall (Montrul, 2010).

Because the acceptability task used a a rating scale, the results were analyzed using the *clmm* (cumulative link mixed model) function in the “ordinal” package (Christensen, 2015) using R (version 1.1.453 for Mac OS X, R Development Core Team, 2014). *Clmm*s were performed on the ordinary-scaled data to model both participant- and item-variability (Agresti, 2002). The raw scores were entered as primary outcome measures (i.e., item ratings per participant and condition) into the statistical analyses. Markedness ([+DOM] vs. [-DOM]) and animacy of the object (animate vs. inanimate) were both fixed effects. Subject and item were included as random effects not standardized because *clmm*s take inter-participant variation into consideration. *Clmm*s were performed separately for each type of sentence (SVO vs. VSO), and the results obtained for each sentence type are discussed below. Proficiency scores were included as covariates to assess the extent to which proficiency of the participants affected their performance.

### SVO sentences

**Figure 5:**
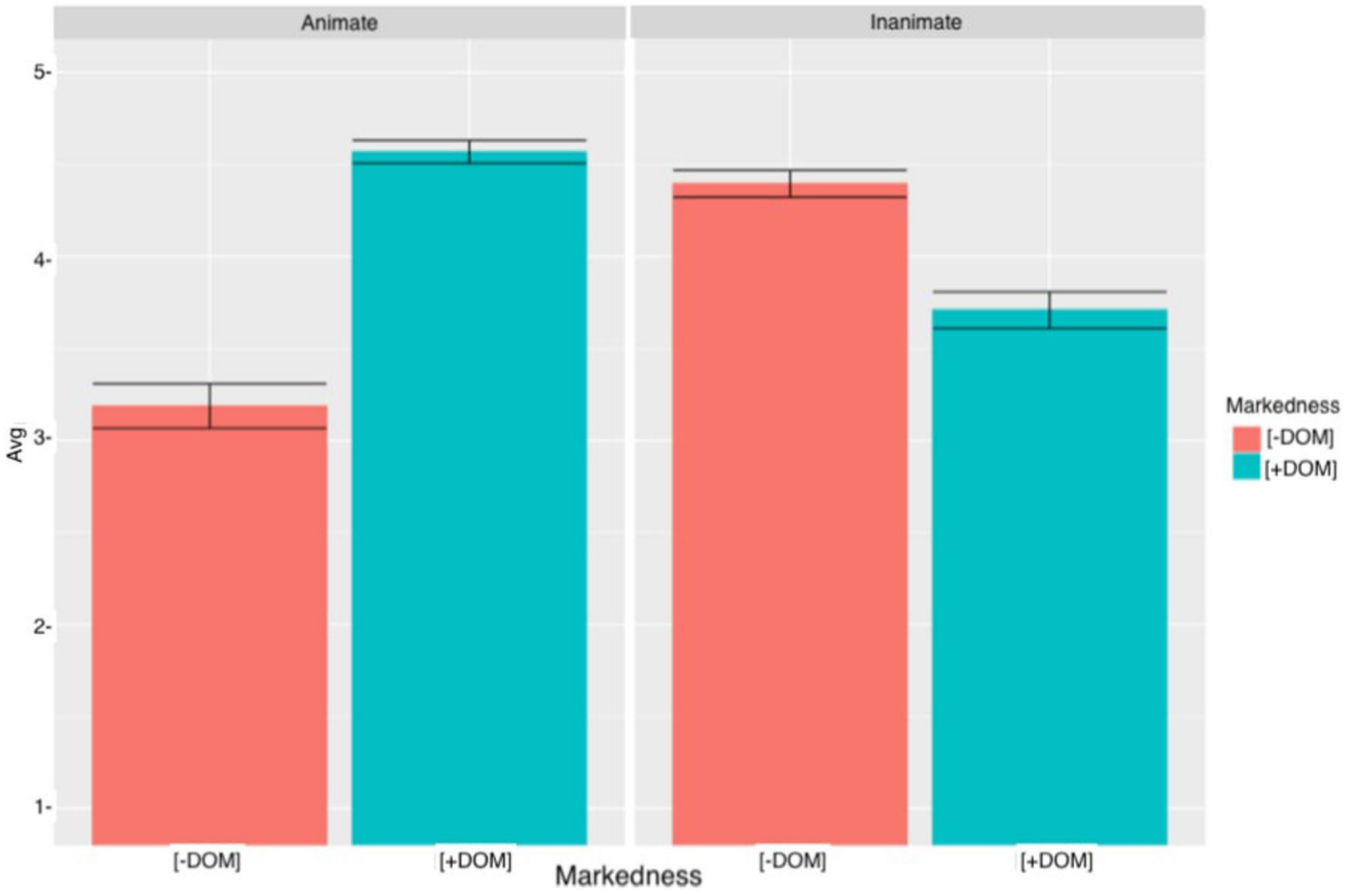
Heritage speakers’ mean acceptability scores and errors bars (95% CI) for SVO sentences.

Figure 5 shows that with animate objects, heritage speakers accepted more grammatical sentences with DOM (M = 4.57, SD = 0.97) than ungrammatical sentences with DOM omission (M = 3.19, SD = 1.47). However, heritage speakers seemed unsure about the rejection of sentences with unmarked animate objects. With inanimate objects, heritage speakers rejected more the use of DOM (M = 3.71, SD = 1.41) than the omission of DOM (M = 4.4, SD = 1.01). However, there was a lot of variation among heritage speakers’ answers, especially with rejection of DOM omission with animate objects and the extension of DOM to inanimate objects. These patterns suggest that while some participants rejected unmarked animate objects and marked inanimate objects, others accepted them.

**Figure 6:**
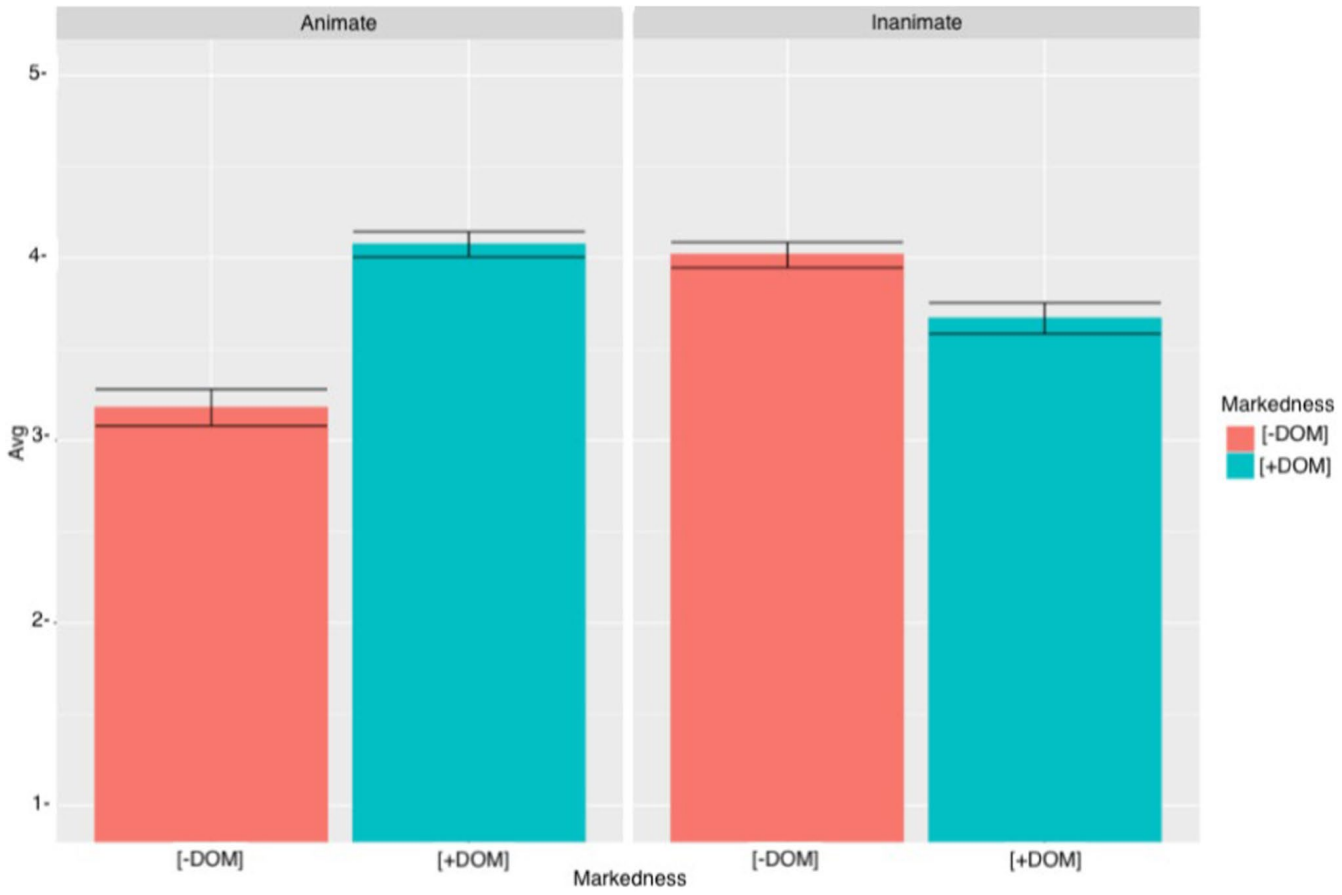
L2 learners’ mean acceptability scores and errors bars (95% CI) for SVO sentences.

Figure 6 shows the results obtained by the L2 learners. Similar to the heritage speakers, the L2 learners accepted sentences with DOM and animate objects DOM (M = 4.07, SD = 1.21) more than the ungrammatical sentences with DOM omission and animate objects (M = 3.18, SD = 1.42). As for the sentences with inanimate objects, L2 learners rejected the sentences with DOM (M = 4.02, SD = 1.11) more often than the sentences with DOM omission (M = 3.67, SD = 1.33). Among the L2 learners there was also a lot of variation which suggests that participants had different judgments about the acceptance/rejection of these sentences. The cumulative link mixed model revealed a significant MARKEDNESS effect (*β* = 1.38, SE = 0.18, *t* = 7.65, *p* < 0.0001) and a significant ANIMACY effect (*β* = 1.19, *SE* = 0.18, *t* = 6.59, *p* < 0.0001). There was also a significant MARKEDNESS*ANIMACY interaction (*β* = −1.92, SE = 0.24, *t* = −7.73, *p* < 0.0001), a significant MARKEDNESS*GROUP interaction (*β* = 1.43, SE = 0.26, *p* < 0.0001), a significant ANIMACY * GROUP interaction (*β* = 1.34, SE = 0.28, *t* = 4.70, *p* < 0.0001) and a significant MARKEDNESS*ANIMACY*GROUP interaction (*β* = −2.10, SE = 0.39, *t* = −5.35 *p* < 0.0001). *Post hoc* analyses for the three-way interaction revealed that the heritage speakers (*β* = −1.38, SE = 0.18, *t* = −7.65, *p* < 0.0001) and the L2 learners (*β* = −2.73, SE = 0.23, *t* = −11.76, *p* < 0.0001) accepted sentences with DOM and animate objects significantly more than sentences with animate objects and DOM omission. Moreover, both groups rejected sentences with inanimate objects and DOM significantly more than sentences with animate objects and DOM (*β* = 0.54, SE = 0.16, *t* = 3.19, *p* = 0.03) (*β* = 1.30, SE = 0.20, *t* = 6.23, *p* < 0.0001). Interestingly, when comparing sentences with unmarked animate objects to sentences with marked inanimate objects, there was a significant effect for heritage speakers (*β* = −0.73, SE = 0.20, *t* = −3.53, *p* = 0.009) and for L2 learners (*β* = −0.65, SE = 0.18, *t* = −3.62, *p* = 0.006). These results suggest that, for heritage speakers and for L2 learners, there is more of a tendency to expand DOM to inanimate objects than to omit DOM with animate objects. Finally, when comparing sentences with marked animate objects to sentences with unmarked inanimate objects, there was not a significant effect for the heritage speakers (*β* = 0.19, SE = 0.16, *t* = 0.16, *p* = 0.94), but the difference was significant for the L2 learners (*β* = 0.69, SE = 0.22, *t* = 3.07, *p* = 0.04). The L2 learners accepted marked animate objects significantly more than unmarked objects. Proficiency was not significant, which suggests that participants’ proficiency did not have an effect on their acceptability ratings.

Following previous studies, heritage speakers and L2 learners were expected to accept sentences with animate objects and DOM omission. Results showed that heritage speakers and L2 learners showed some acceptance of unmarked animate objects, but both groups still rated sentences with animate objects and DOM significantly higher. Nevertheless, as Figures 5, 6 show, there was a great deal of variation among heritage speakers’ and L2 learners’ responses, and while some participants appeared to reject sentences with animate objects and no DOM, others accepted them. As for the sentences with inanimate objects, participants were expected to accept sentences with DOM omission and reject sentences with DOM. While results revealed a significant effect between sentences with DOM and sentences with DOM omission, participants did not always reject sentences with DOM, and there was notable variation among their answers. Moreover, heritage speakers and L2 learners preferred the extension of DOM to inanimate objects over the omission of DOM with animate objects. Because PROFICIENCY did not turn out to be significant (*β* = 0.006, SE = 0.01, *t* = 0.03, *p* = 0.97), it appears that participants’ proficiency does not have an effect on their judgments.

### VSO sentences

**Figure 7:**
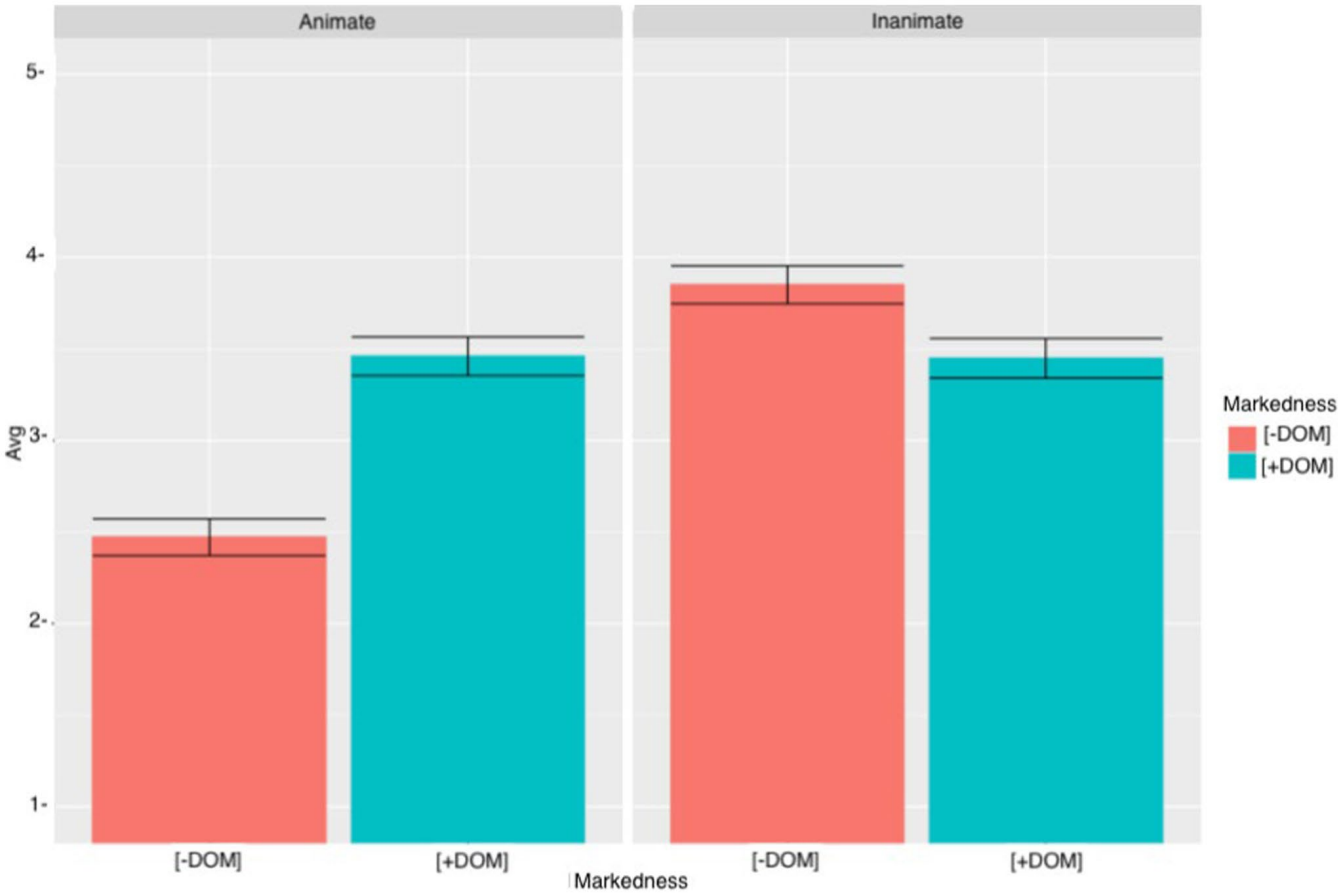
Heritage speakers means acceptability scores and errors bars (95% CI) for VSO sentences.

Figure 7 shows the results obtained for the heritage speakers. With animate objects, heritage speakers rated the sentences with DOM (M = 3.46, SD = 1.39) higher than the sentences with DOM omission (M = 2.47, SD = 1.35). However, when accepting sentences with inanimate objects, heritage speakers accepted ungrammatical DOM omission (M = 3.85, SD = 1.42) more than the use of DOM (M = 3.45, SD = 1.49).

**Figure 8:**
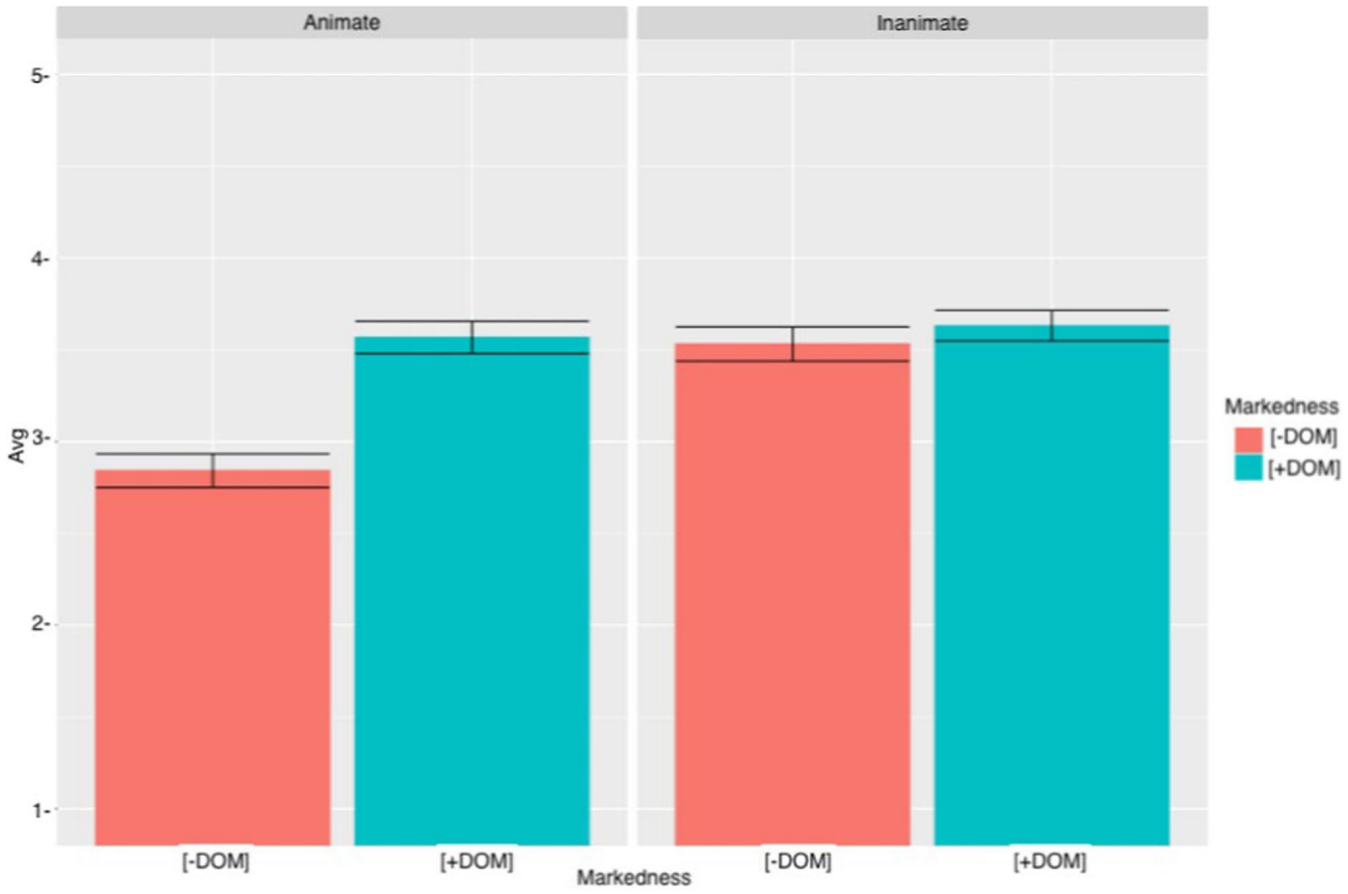
L2 learners’ mean acceptability scores and errors bars (95% CI) for VSO sentences.

Regardless of the type of the object or the use of DOM, there was variation on heritage speakers’ answers regarding the acceptance of these sentences. Similar to the heritage speakers, when judging the sentences with animate objects, the L2 learners rated the sentences with DOM (M = 3.57, SD = 1.36) higher than the sentences with DOM omission (M = 2.84, SD = 1.39) (see Figure 8). As for the sentences with inanimate objects, contrary to what it was hypothesized, L2 learners preferred the sentences with DOM (M = 3.63, SD = 1.28) over the sentences with DOM omission (M = 3.53, SD = 1.42). Overall, there was a lot of variation in L2 learners’ answers. The cumulative link mixed model for VSO sentences revealed a significant MARKEDNESS effect (*β* = 1.40, SE = 0.20, *t* = −6.80, *p* < 0.0001) and a significant ANIMACY effect (*β* = 2.24, SE = 0.21, *t* = −10.51, *p* < 0.0001). There was also a significant MARKEDNESS * ANIMACY interaction (*β* = −2.19, SE = 0.29, *t* = −7.45, *p* < 0.0001), a significant ANIMACY *GROUP interaction (*β* = −1.21, SE = 0.27, *t* = −4.40, *p* < 0.0001) and a significant MARKEDNESS*ANIMACY*GROUP (*β* = 1.20, SE = 0.38, *t* = 3.13, *p* < 0.001). Tukey’s multiple comparison test for the three-way interaction revealed a significant effect when comparing sentences with animate objects with and without DOM for heritage speakers (*β* = −1.40, SE = 0.20, *t* = −6.80, *p* < 0.0001) and L2 learners (*β* = −1.06, SE = 0.17, *t* = −5.99, *p* < 0.0001). However, when comparing sentences with inanimate objects with DOM and without DOM, there was only a significant effect for heritage speakers (*β* = 0.79, SE = 0.20, *t* = −3.82, *p* < 0.0001), but not for L2 learners (*β* = −0.07, SE = 0.17, *t* = −0.43, *p* = 0.99). Therefore, only the heritage speakers rejected the extension of DOM to inanimate objects. When comparing unmarked animate objects to marked inanimate objects, there was a significant effect for heritage speakers (*β* = −1.45, SE = 0.20, *t* = −7.09, *p* < 0.0001) and for L2 learners (*β* = −0.07, SE = −1.11, *t* = −6.27, *p* < 0.0001). These results suggest that heritage speakers and L2 learners prefer DOM with inanimate objects over the omission of DOM with animate objects. However, when comparing sentences with marked animate objects to sentences with unmarked inanimate objects there was a significant effect for heritage speakers (*β* = −0.84, SE = 0.20, *t* = −4.03, *p* = 0.011), but the difference was not significant for the L2 learners (*β* = 0.03, SE = 0.17, *t* = 0.19, *p* = 1.00). Heritage speakers, but not L2 learners, accepted unmarked inanimate objects significantly more than marked animate objects. Finally, proficiency did not turn out to be significant (*β* = 0.01, SE = 0.02, *t* = 0.75, *p* = 0.45), which suggests that participants’ proficiency did not have an effect on their judgments.

#### Summary of results

As hypothesized, heritage speakers and L2 learners did not completely reject the omission of DOM with animate objects in any of the contexts. In most cases, heritage speakers and L2 learners appeared to be undecided when judging unmarked animate objects. However, heritage speakers and L2 learners with SVO and VSO rejected the omission of DOM with animate objects more than the use of DOM with inanimate objects. In fact, results obtained from the sentences containing inanimate objects were unexpected, as neither the heritage speakers nor the L2 learners showed a strong rejection of the use of DOM with inanimate objects. Moreover, proficiency did not appear to be significant in any of the analyses, and thus, contrary to what was predicted (Montrul, 2010), participants with higher proficiency did not behave differently than participants with lower proficiency.

It was also hypothesized that word order would have an effect on participants’ judgments. Results partially support this hypothesis as word order had an effect only on sentences with inanimate objects and only with L2 learners: for sentences with a non-canonical word order, L2 learners’ judgment did not differ between sentences with DOM and sentences with DOM omission. Therefore, results suggest that L2 learners sometimes accepted the use of DOM with inanimate objects. Participants may accept DOM with inanimate objects due to an overgeneralization error. However, because they did not accept the use of DOM with inanimate objects in sentences with a canonical word order, the fact that they accept DOM extension in VSO sentences may be more related to the word order of these sentences. When reading sentences with non-canonical word order, participants may find these sentences unnatural and pay less attention to the use of DOM. In fact, heritage speakers’ and L2 learners’ ratings were overall lower with sentences following a non-canonical word than with sentences following a canonical word order (SVO).

Heritage speakers and L2 learners showed some DOM retraction in both the oral tasks and the AJT. However, results suggest an opposite production-comprehension asymmetry: while heritage speakers showed more DOM omission in the AJT than in the oral tasks, L2 learners showed more DOM omission in the oral tasks than in the AJT. The next step is to analyze their online comprehension. It seems that heritage speakers integrate DOM into their processing, and that is why they are able to almost always produce it. As for the L2 learners, following the MSIH, they may also integrate DOM into their online comprehension, but due to production specific problems brought on by communicative pressure, DOM is not part of their productive knowledge.

### Reading comprehension task with eye-tracking

The aim of this task was to test heritage speakers and L2 learners’ sensitivity to DOM while reading. This task measured participants’ sensitivity to DOM during reading comprehension. The basic assumption in reading tasks with eye-tracking is that participants’ eye movements are slower (fixed on the target longer) or produce more regressions (return to a specific region) when reading something unexpected. For example, when presented with sentences such as * *Juan vio el policía* ‘Juan saw the policeman’ and *Juan vio al policía* ‘Juan saw DOM-the policeman’, participants are expected to take longer to read the first sentence or produce more regressions if they are aware that animate and specific objects must be marked with DOM.

Participants read sentences that varied by MARKING ([+DOM] vs. [**-**DOM]) and animacy of the object (animate vs. inanimate) and word order (SVO and VOS). Table 6 shows examples of the sentences used in this task.

**Table 6:** Sample sentences used in the eye-tracking task.

Direct object	[+DOM]	[−DOM]
Animate	El actor liberó al compañero con su llave.	*El actor liberó el compañero con su llave.
	‘The actor freed DOM the companion with his key.’	‘The actor freed the companion with his key.’
Inanimate	*El joven movió al sofá a la calle para dormir.	El joven movió el sofá a la calle para dormir.
	‘The young man moved DOM the sofa to the street to sleep.’	‘The young man moved the sofa to the street to sleep.’

Notice in (9) that all objects (e.g., *compañero*, *sofá*) were singular and masculine objects with the case marker merged with the article (a + el = al). In this way, it is possible to compare ‘el’ versus ‘al’ because they are segments of equal length. All sentences were between 8 and 9 words in length and were preceded by a prepositional phrase because it is recommended to avoid having the critical, or even the spillover, region at the beginning of a sentence in eye-tracking with text tasks. Fixations tend to be longer at the beginning of a sentence and people often make corrective saccades (Rayner, 1979; Heller,1982). All experimental sentences and fillers were followed by comprehension questions about the content of the sentences. The fillers used in this task were very similar to the filler sentences used in the AJT. The comprehension questions had nothing to do with agent/patient relationships so as not to direct the participants’ attention to the experimental manipulation, as in (9).

(9) El actor liberó al compañero con su llave.
‘The actor released his partner with his key.’
*¿Qué usó el actor?*
‘What did the actor use?
A) Una llave B) Unas tijeras.
a key’‘a pair of scissors’.

Based on previous studies, heritage speakers and L2 learners were expected to show no sensitivity to DOM with animate objects with canonical word order sentences (Arechabaleta-Regulez, 2016; Jegerski, 2018; Jegerski and Sekerina, 2019). Therefore, participants were expected to produce comparable reading times when reading sentences with marked animate objects than with sentences with DOM omission. As for sentences with inanimate objects, heritage speakers and L2 learners were expected to show sensitivity to DOM (Jegerski, 2018). Therefore, they were expected to produce longer reading times and more regressions with marked than with unmarked inanimate objects. Moreover, word order was hypothesized to play a role in participants’ sensitivity to DOM. If Heritage speakers and L2 learners showed some sensitivity to DOM, it would be more prominent with non-canonical word order sentences than sentences with canonical word order (Arechabaleta-Regulez, 2016; Jegerski and Sekerina, 2019). However, proficiency was expected to play a role and only those participants with a high proficiency in Spanish are expected to show DOM sensitivity, particularly with objects in sentences with non-canonical word order.

## Reading task with eye-tracking

### Results

Eye movement data was analyzed off-line to identify fixations and saccades using the DataViewer software package (SR Research Ltd., version 1.11.1). Data for the reading task with eye-tracking was analyzed with the *lmer* (linear mixed effect regression) function in the lme4 package (Bates et al., 2015) using R (version 1.1.453 for Mac OS X, R Development Core Team, 2014) for every eye movement measurement. For all analyses, reading times were the dependent variable while markedness ([+DOM] vs. [-DOM]), animacy of the object (animate vs. inanimate) and group (heritage speakers vs. L2 learners) were all fixed effects. Subject and item were both included as random effects. Proficiency scores were included as covariates to assess the extent to which proficiency affected participants’ processing. When significant interactions were found, a Tukey’s multiple comparison *post hoc* test was performed with *lmeans* package to conduct multiple pairwise comparisons of the fixed variables and their interactions. To ensure that the descriptive and statistical analyses included only sentences that participants understood, sentences with incorrect responses to the post-stimulus comprehension questions were excluded from the analyses. Also, all fixations shorter than 80 ms and longer than 1,200 ms were excluded (Rayner, 1979). In total, this excluded 15.1% of the data (see Table 7).

**Table 7:** Mean accuracy scores for the comprehension questions.

	Heritage Speakers	L2 learners
Correct	91.20%	89.4%
Incorrect	8.8%	10.6%

Table 7 shows that, overall, heritage speakers were more accurate than the L2 learners with the post-stimulus comprehension questions; however, there was not a significant GROUP comparison (*β* = 10.77, SE = 9.33, *t* = 1.01, *p* = 0.22). Results for each type of sentence are discussed in the following subsections. Each discussion begins with a table displaying the mean reaction times in milliseconds as well as the standard errors for each of the 5 reading times and in each of the 4 regions: the Critical Region, Region 4, Region 5 and Region 6. Notice in (10) that all sentences were divided into 8 different regions (R) of interest. While the Critical Region (CR) was Region 3 (the region in which DOM is either used or omitted), processing effects could occur after the Critical Region (spillover effect) (Arechabaleta-Regulez, 2016). Therefore, not only the CR, but also Region 4(R4), Region 5(R5) and Region 6 (R6) were analyzed. Five reading times were analyzed: *second pass reading times*, *total reading times*, *number of regressions out* and *number of regression in*. Second pass reading times were analyzed to measure the time participants spend in each region when re-reading the sentence. Total reading times were run to measure the total time participants spent in each region of the sentence. Finally, number of regressions out and number of regressions in were calculated for each sentence. While number of regressions out refers to the number of times a region was exited (with an eye regression) to a previous region, number of regressions in refers to the number of times a region was entered (with an eye regression) from a later region. Only significant effects and significant interactions are discussed.

(10)

El actor libe ró al compañero von su llave

R1 R2 CR R4 R5 R6 R7

### SVO sentences

Supplementary Tables 8, 9 show the mean reaction times in milliseconds as well as the standard deviation for each of the 5 reading times and in each of the 4 regions analyzed for SVO sentences for heritage speakers and L2 learners, respectively.

#### Total reading times

In Region 4, there was a significant MARKEDNESS*ANIMACY interaction (*β* = 89.05, SE = 42.50, *t* = 2.09, *p* = 0.03). As Supplementary Table 10 shows, when reading sentences with animate objects, heritage speakers and L2 learners needed more time to read sentences with DOM omission than with DOM; however, when reading sentences with inanimate objects, heritage speakers and L2 learners needed more time to read sentences with DOM than with DOM omission. Because the animacy of the object caused opposite effects to the use or omission of DOM, the result is an interaction between the two factors without a main effect (known as a crossover interaction). Therefore, it appears that both groups were sensitive to the omission of DOM with animate objects and to the use of DOM with inanimate objects. In Region 6, there was a significant ANIMACY* GROUP interaction (*β* = −89.23, SE = 39.4, *t* = −2.26, *p* = 0.02). Tukey’s multiple comparison test revealed a significant difference between the reading times produced by L2 learners with animate and inanimate objects (*β* = 62.99, SE = 23.07, *t* = 2.73, *p* = 0.03). As Supplementary Table 11 shows, both groups produced longer reading times with animate than with inanimate objects.

#### First pass reading times

In Region 5, there was a significant MARKEDNESS* GROUP interaction (*β* = 25.62, SE = 12.96, *t* = 1.97, *p* = 0.04). Tukey’s test did not reveal any significant comparisons. Heritage speakers and L2 learners behaved differently with regard to the use or omission of DOM. Heritage speakers took longer to read sentences without DOM, while L2 learners produced longer reading times with DOM-marked objects (crossover interaction) (see Supplementary Table 12).

#### Second pass reading times

There was a significant MARKEDNESS*ANIMACY interaction in Region 4 (*β* = 96.02, SE = 41.89, *t* = 2.29, *p* = 0.02). Tukey’s multiple comparison tests did not reveal any significant comparisons (crossover interaction). As Supplementary Table 13 shows, participants took longer to read unmarked objects than marked objects with sentences containing animate objects; however, for sentences containing inanimate objects, participants needed more time to read DOM-marked objects than unmarked objects. In Region 6, there was a significant GROUP effect (*β* = 97.9, SE = 35.85, *t* = 2.73, *p* = 0.006) and a significant ANIMACY* GROUP interaction (*β* = −85.17, SE = 37.43, *t* = −2.27, *p* = 0.02). Tukey’s test revealed a significant comparison between the reading times produced by the heritage speakers and the L2 learners when reading sentences with animate objects (*β* = −87.60, SE = 30.96, *t* = −2.82, *p* = 0.02). There was also a significant comparison between the heritage speakers’ reading times when reading sentences with inanimate objects and the L2 learners’ reading times when reading sentences with animate objects (*β* = −100.46, SE = 32.96, *t* = −3.04, *p* = 0.01). Finally, the analysis found a significant difference for the L2 learners with sentences with animate and inanimate objects (*β* = 65.94, SE = 21.00, *t* = −3.13, *p* = 0.01). As Supplementary Table 14 shows, heritage speakers and L2 learners took longer to read sentences with animate than with inanimate objects.

#### Number of regressions in

Number of regressions in revealed a significant ANIMACY effect (*β* = −9.57, SE = 4.74, *t* = −2.01, *p* = 0.04), a significant PROFICIENCY effect (*β* = 4.88, SE = 2.48, *t* = 1.96, *p* = 0.05) and a significant MARKEDNESS*ANIMACY interaction (*β* = 1.24, SE = 6.72, *t* = 1.84, *p* = 0.05) in Region 4. Tukey’s multiple comparison test did not reveal any significant differences. With animate objects, heritage speakers and L2 learners both produced more regressions in with when animate objects were unmarked. However, they produced more regressions in with sentences that contained DOM-marked inanimate objects (see Supplementary Table 15). Proficiency turned out to be significant because participants with a lower proficiency produced more regressions overall than participants with a higher proficiency. In Region 5, there was a significant ANIMACY*GROUP interaction 2 (*β* = 252.35, SE = 91.85, *t* = 2.75, *p* = 0.005). As Supplementary Table 16 shows, heritage speakers produced more regressions in with animate objects, while L2 learners did so with inanimate objects. In Region 6, there were not significant effects or significant interactions.

#### Number of regressions out

Number of regressions out revealed a significant ANIMACY*GROUP interaction (*β* = 8.98, SE = 4.50, *t* = 1.99, *p* = 0.04) and a significant MARKEDNESS*ANIMACY*GROUP interaction (*β* = −1.17, SE = 6.38, *t* = −1.83, *p* = 0.05 in Region 4. Tukey’s multiple comparison test did not reveal any significant comparisons. As Supplementary Table 17 shows, both heritage speakers and L2 learners produced more regressions out with unmarked animate objects. As for inanimate objects, heritage speakers produced more regressions out with DOM-marked objects, while the L2 learners with unmarked objects. In Region 6, there was a significant ANIMACY*GROUP interaction (*β* = 1.29, SE = 6.43, *t* = 2.01, *p* = 0.04) and a significant MARKEDNESS*ANIMACY*GROUP interaction (*β* = −1.17, SE = 6.38, *t* = −1.74, *p* = 0.05). Tukey’s test did not reveal any significant comparisons for either of the interactions. As Supplementary Table 18 shows, when reading sentences with animate objects, heritage speakers, but not L2 learners, produced more regressions out with sentences with unmarked objects. With inanimate objects, heritage speakers and L2 learners both regressed out with DOM-marked objects.

#### Sum of skipped targets

In total, heritage speakers skipped DOM or the determiner ‘el’ 10% of the time and L2 learners 12%. There was not a significant GROUP effect (*β* = −0.03, SE = 0.02, *t* = −1.68, *p* = 0.54).

Reading times for SVO sentences suggest that heritage speakers and L2 learners were more sensitive to the omission of DOM with animate objects than previously expected. Heritage speakers and L2 learners were also sensitive to the extension of DOM to inanimate objects as previously suggested (Jegerski, 2018). With total reading times, with first pass reading times, and with regressions in, there was a significant MARKEDNESS*ANIMACY interaction in region 4, as heritage speakers and L2 learners produced longer reading times or more regressions with unmarked animate objects than with marked animate objects and with marked inanimate objects than with unmarked inanimate objects. However, in later regions, it is important to note that heritage speakers seemed to be affected by DOM omission regardless of the animacy of the object, while L2 learners were affected by DOM regardless of the animacy of the object. Finally, proficiency was only significant with regressions in, as participants with a lower proficiency produced more regressions than participants with a higher proficiency.

### VSO sentences

The mean reaction times in milliseconds and the standard deviation for the 7 reading times and for all the 4 regions are represented in Supplementary Table 19 for the heritage speakers and in Supplementary Table 20 for the L2 learners.

#### Total reading times

Total Reading times did not show any significant effects or any significant interactions in any of the 4 regions that were analyzed. Therefore, with VSO sentences, the use or non-use of DOM did not cause any processing difficulties for the heritage speakers or for the L2 learners.

#### First pass reading times

First pass reading times revealed a significant GROUP effect (*β* = −38.57, SE = 13.69, *t* = −2.81, *p* = 0.005) in the Critical Region. In Region 4, there was a significant MARKEDNESS*GROUP interaction (*β* = −23.62, SE = 12.94, *t* = −1.82, *p* = 0.05). Tukey’s multiple comparison test did not reveal any significant comparisons. Heritage speakers and L2 learners reacted differently to the use or omission of DOM. Regardless of the animacy of the object, heritage speakers produced longer reading times when sentences with DOM, while L2 learners produced longer reading times with sentences without DOM (see Supplementary Table 21). In Region 5, there was also a significant ANIMACY*GROUP interaction (*β* = −2.51, SE = 1.38, *t* = −1.82, *p* = 0.04). As Supplementary Table 22 shows, while heritage speakers needed more time to read sentences with inanimate objects, L2 learners needed more time to read sentences with animate objects.

#### Second pass reading times

In the Critical Region (*β* = 105.65, SE = 43.04, *t* = 2.45, *p* = 0.01) and in Region 4 (*β* = 92.25, SE = 45.14, *t* = 2.04, *p* = 0.04), there was a significant GROUP effect. Overall, heritage speakers were faster readers than L2 learners. However, there were not any significant effects or significant interactions.

#### Number of regressions in

In the Critical Region, there was a significant MARKEDNESS*ANIMACY interaction (*β* = 1.15, SE = 6.51, *t* = 1.76, *p* = 0.04) and a significant MARKEDNESS*ANIMACY*GROUP interaction (*β* = −1.64, SE = 9.11, *t* = −1.80, *p* = 0.03). Tukey’s multiple comparison test did not reveal any significant comparisons in any of the interactions, as participants reacted to the use or omission of DOM differently (see Supplementary Table 23). When reading sentences with animate objects, heritage speakers produced more regressions in with sentences that omitted DOM than with sentences containing DOM. As for the L2 learners, they produced more regressions in with sentences with DOM than with sentences that omitted DOM. As for sentences with inanimate objects, heritage speakers produced more regressions in with sentences with DOM, while L2 learners produced more regression in with sentences without DOM. In Region 6, there was a significant MARKEDNESS*ANIMACY*GROUP interaction (*β* = −1.82, SE = 1.01, *t* = −1.80, *p* = 0.04). In this region, Tukey’s test did not reveal any significant effects. However, estimated marginal means showed the same trend as in the critical region. When reading sentences with animate objects, heritage speakers produced more regressions in with sentences without DOM, while L2 learners produced more regressions in with sentences with DOM. As for sentences with inanimate objects, heritage speakers produced more regressions in with sentences with DOM, while L2 learners produced more regressions in with sentences without DOM (see Supplementary Table 24).

#### Number of regressions out

Number of regressions out revealed a significant MARKEDNESS* GROUP interaction (*β* = −1.36, SE = 5.41, *t* = −2.52, *p* = 0.01) and a significant MARKEDNESS*ANIMACY*GROUP interaction (*β* = 1.46, SE = 7.47, *t* = 1.95, *p* = 0.05) in the Critical Region. Tukey’s multiple comparison test revealed an almost significant comparison between the regressions out produced by the L2 learners when reading sentences with animate objects and DOM omission and sentences with animate objects and DOM (*β* = 0.11, SE = 0.04, *t* = 2.99, *p* = 0.06). As Supplementary Table 25 shows, when reading sentences with animate objects, heritage speakers and L2 learners produced more regressions out with unmarked objects than with marked objects. As for sentences with inanimate objects, heritage speakers produced more regressions with marked objects, while L2 learners produced more regressions out with sentences that omitted DOM (see Supplementary Table 25). In Region 4, there was also a significant MARKEDNESS*ANIMACY*GROUP interaction (*β* = −1.87, SE = 9.25, *t* = −2.02, *p* = 0.04). For this region, Tukey’s test did not reveal any significant comparisons. As Supplementary Table 26 shows, heritage speakers produced more regressions out with unmarked animate objects and with marked inanimate objects than with marked animate objects and unmarked inanimate objects, respectively. L2 learners on the other hand, produced more regressions out with unmarked objects regardless of the animacy of the object. There was also a significant PROFICIENCY effect in Regions 5 (*β* = −4.39, SE = 2.41, *t* = −1.82, *p* = 0.07) and 6 (*β* = −7.44, SE = 3.42, *t* = −2.17, *p* = 0.03).

#### Sum of skipped targets

Overall, heritage speakers skipped the Critical Region 16% of the time and L2 learners 19% of the time. The pairwise comparison did not show a significant GROUP effect (*β* = −0.04, SE = 0.02, *t* = −2.37, *p* = 0.16).

Overall, reading times for sentences with VSO word order showed mixed results regarding the sensitivity to DOM with animate and inanimate objects by the heritage speakers and the L2 learners. The heritage speakers, as suggested by total reading times and second pass reading times, produced longer reading times with marked animate objects than with unmarked animate objects in early regions (R2, R3, R4 and R5); however, in later regions (R6, R7 and R8) they showed the opposite pattern: they produced longer reading times with sentences without DOM than with sentences with DOM. These results may suggest that their sensitivity to DOM omission with animate objects happened only in later regions as a spillover effect. Regressions in and regressions out supported this possibility, as the heritage speakers showed sensitivity to the omission of DOM with animate objects: heritage speakers produced more regressions (in and out) with unmarked than with marked animate objects. As for sentences with inanimate objects, sensitivity to the use or omission of DOM was only perceived by regressions in and regressions out: the heritage speakers produced more regressions (in and out) with marked inanimate objects than with unmarked inanimate objects. The L2 learners showed less sensitivity to the use or omission of DOM regardless of the animacy of the object. Some type of DOM sensitivity was only perceived by regressions in and regressions out and only for animate objects. The L2 learners tended to produce more regressions (in and out) with unmarked animate objects than with marked animate objects.

#### Summary of results

The aim of this task was to test participants’ sensitivity to DOM while reading. With SVO sentences, heritage speakers and L2 learners were not expected to show DOM sensitivity. However, with non-canonical sentences participants were expected to show some sensitivity (Arechabaleta-Regulez, 2016) by producing longer reading times with unmarked animate objects than with marked animate objects and with marked inanimate objects than with unmarked inanimate objects. Heritage speakers and L2 learners were expected to rely on processing mechanisms (word order) in their stronger language (English) to comprehend these sentences instead of object marking. However, with non-canonical sentences (VSO), participants were expected to rely more on DOM and thus show more sensitivity to it, as it was more relevant for comprehending these sentences. Finally, participants with a higher proficiency were expected to show more DOM sensitivity with both, animate and inanimate objects.

Contrary to what was hypothesized, the heritage speakers and the L2 learners showed more DOM sensitivity with SVO sentences than with VSO sentences. Sensitivity to DOM with SVO sentences happened with later measures, as both groups of bilinguals produced longer reading times and/or more regressions with sentences with unmarked animate objects than with marked animate objects and with marked inanimate objects than with unmarked inanimate objects. As for VSO sentences, heritage speakers appeared to show more sensitivity to DOM than the L2 learners. However, sensitivity to DOM by the heritage speakers was only observable with regressions (in and out) and in later regions of the sentences. Thus, sensitivity did not appear squarely in the Critical Region but as a post-critical effect. The L2 learners, on the other hand, did not show sensitivity to DOM with either animate or inanimate objects, contrary to what was hypothesized. Finally, while participants were expected to skip DOM more often with SVO than with VSO sentences, results did not support this hypothesis. In fact, heritage speakers and L2 learners skipped DOM most with VSO sentences.

## Discussion and conclusion

The purpose of this study was to analyze the production, acceptability and online comprehension of Spanish DOM by two groups of bilingual speakers living in the U.S.: heritage speakers and L2 learners. While previous studies have reported these two groups of bilingual speakers tend to omit DOM with animate objects (Farley and McCollam, 2004; Guijarro-Fuentes and Marinis, 2007; Bowles and Montrul, 2008, 2009; Montrul, 2010, 2014; Montrul and Sánchez-Walker, 2013; Arechabaleta-Regulez, 2014; Jegerski, 2018; Jegerski and Sekerina, 2019), these studies have usually examined production, acceptability or online comprehension of DOM in isolation. Few, if any, have compared all three of these aspects with the same group of speakers. Therefore, this study employed tasks designed to elicit data related to all of participants’ production, acceptability and online comprehension. The same group of heritage speakers and the same group of L2 learners completed all the tasks.

First, the oral tasks were used to analyze heritage speakers’ and L2 learners’ production of DOM. It was predicted that both groups would show significant DOM omission with animate objects, but that heritage speakers would show less DOM omission overall (Montrul, 2010). In addition, proficiency in Spanish was expected to play an important role. Lastly, heritage speakers’ and L2 learners’ production of DOM was hypothesized to depend on the type of task because the narrative task was seen as a more implicit task than the elicitation task. Results showed that: (1) as predicted, both groups omitted DOM with animate objects; (2) L2 learners showed more cases of DOM omission than heritage speakers; (3) proficiency played a significant role, as participants with a higher proficiency in Spanish marked animate objects more than participants with a lower proficiency; (4) proficiency was more significant for L2 learners than for heritage speakers; and (5) type of task indeed had an effect but not the effect that was expected. Results partially supported the hypotheses, as only the heritage speakers were affected by the type of task. The heritage speakers showed more DOM retraction in the elicitation task than in the oral task and also produced significantly more DOM omission to inanimate objects in the elicitation task than in the narrative task. However, contrary to what was hypothesized, the L2 learners did not show less DOM omission in the elicitation task than in the narrative task. In fact, the L2 learners behaved very similarly in the two oral tasks.

Second, the aim of the AJT was to analyze participants’ knowledge of DOM. Following previous studies, both heritage speakers and L2 learners were expected to accept DOM omission with animate objects and to reject the use of DOM with inanimate objects. Additionally, word order was manipulated in the AJT. It was hypothesized that participants would have to pay closer attention to the use or omission of DOM in sentences following a non-canonical word order and thus might show less DOM attrition with VSO sentences. Finally, proficiency was expected to play a role. Results showed that: (1) as predicted, heritage speakers and L2 learners had difficulty rejecting sentences with DOM omission and animate objects; (2) surprisingly, both heritage speakers and L2 learners also had a hard time rejecting sentences with DOM extension to inanimate objects, especially L2 learners. In fact, the acceptability ratings given by L2 learners to VSO sentences with DOM did not significantly differ from those given to sentences with DOM omission; (3) results did not support the hypothesis on the effects of word order. Participants did not integrate DOM more in sentences following a non-canonical word order. Overall, SVO sentences were rated higher than VSO sentences. The rejection of VSO sentences may be due to the fact that they follow a non-canonical word order. Thus, heritage speakers and L2 learners may not be as familiar with these sentences and could perceive them as less acceptable regardless of the use of DOM or the animacy of the objects. Finally, (4) proficiency did not turn out to be significant. Therefore, heritage speakers and L2 learners’ performance did not depend on their proficiency in Spanish.

Considered together, the results obtained in the oral tasks and the results obtained in the AJT support the relevance of language experience and practice in language acquisition. Because heritage speakers have acquired DOM orally and implicitly, they relied on implicit knowledge and integrated DOM more in the oral tasks than in the AJT. L2 learners, on the other hand, have acquired DOM in the classroom and most likely *via* metalinguistic explanations. Thus, they applied that explicit knowledge in the AJT but not in the oral tasks (as suggested by the MSIH; Prévost and White, 1999, 2000). Nevertheless, as suggested by the results obtained in the reading task with eye-tracking, L2 learners can still integrate DOM into their online comprehension.

The reading task with eye-tracking aimed to analyze participants’ processing mechanisms to test whether DOM omission is part of their competence. It was hypothesized that heritage speakers would show little sensitivity to unmarked animate objects, at least with sentences following a canonical word order (Jegerski, 2015, 2018; Arechabaleta-Regulez, 2016; Jegerski and Sekerina, 2019). Moreover, heritage speakers and L2 learners were expected to show sensitivity to DOM with inanimate objects regardless of the word order (Jegerski, 2018). Lastly, proficiency was also thought to play an important role, and participants with a higher proficiency were expected to show more DOM sensitivity. Results showed that, contrary to what was predicted, both heritage speakers and L2 learners showed more DOM sensitivity with canonical word order sentences than with non-canonical word order sentences. With SVO sentences, the heritage speakers and the L2 learners showed sensitivity to unmarked animate objects and marked inanimate objects in late reading measures and immediately after the critical region. With VSO sentences, only the heritage speakers showed a degree of sensitivity to DOM omission with animate objects and to the use of DOM with inanimate objects. Sensitivity was only perceived with regressions in and out. Finally, proficiency did not play an important role in the reading mechanisms produced by the participants.

All in all, results showed that DOM variation exists among heritage speakers and L2 learners. Both heritage speakers and L2 learners can integrate DOM into their production, judgments and processing, but they do so inconsistently. Type of task and type of sentence each have an effect on speakers’ use of DOM. These effects were not always the same for both heritage speakers and L2 learners, which corroborates the importance that language experience and language practice have on speakers’ actual use of DOM.

## Data availability statement

The original contributions presented in the study are included in the article/Supplementary material, further inquiries can be directed to the corresponding author.

## Ethics statement

The studies involving human participants were reviewed and approved by University of Illinois at Urbana Champaign. The patients/participants provided their written informed consent to participate in this study.

## Author contributions

BA and SM: methodology and formal analysis. BA: data collection, writing—original draft preparation, and writing—review and editing. SM: supervision and editing. All authors contributed to the article and approved the submitted version.

## Funding

This research was partially funded by the Love Fellowship (UIUC Center for Latin American and Caribbean Studies).

## Conflict of interest

The authors declare that the research was conducted in the absence of any commercial or financial relationships that could be construed as a potential conflict of interest.

## Publisher’s note

All claims expressed in this article are solely those of the authors and do not necessarily represent those of their affiliated organizations, or those of the publisher, the editors and the reviewers. Any product that may be evaluated in this article, or claim that may be made by its manufacturer, is not guaranteed or endorsed by the publisher.

## Footnotes

^1^The notions of definiteness and specificity are both discourse related. The notion of definiteness refers to the state of knowledge shared between the speaker and hearer (or writer and reader), while the notion of specificity refers to the state of knowledge known to the speaker (writer) only (Ionin et al., 2004).
