# Supplementary material for: Production, acceptability, and online comprehension of Spanish differential object marking by heritage speakers and L2 learners
Source: Front Psychol. 2023 Apr 20;14:1106613. doi: 10.3389/fpsyg.2023.1106613 (PMC10157495; doi:10.3389/fpsyg.2023.1106613)
Supplement: Supplementary file 1 [file Data_Sheet_1.docx]

**Table 8:** Reaction times and standard deviations for SVO sentences (HS)

|  | TT | SP | FP | RI | RO |
| --- | --- | --- | --- | --- | --- |
| Region 3 |  |  |  |  |  |
| Animate + DOM | 839.51  (328.48 | 265.61  (124.00) | 573.90  (309.93) | 0.48  (0.50) | 0.13  (0.33) |
| Animate - DOM | 823.74  (330.28 | 254.16  (114.11) | 569.57  (300.79) | 0.50  (0.50) | 0.14  (0.34) |
| Inanimate - DOM | 807.02  (316.19) | 244.52  (101.52) | 562.50  (289.37) | 0.50  (0.50 | 0.16  (0.36) |
| Inanimate +DOM | 839.58  (312.27) | 241.86  (103.91) | 597.72  (294.86) | 0.50  (0.50 | 0.10  (0.30) |
| Region 4 |  |  |  |  |  |
| Animate + DOM | 766.85  (300.77) | 266.48  (117.81) | 500.37  (284.30) | 0.26  (0.44) | 0.24  (0.43) |
| Animate - DOM | 799.55  (326.70) | 258.81  (107.38) | 540.74  (304.75) | 0.33  (0.47) | 0.28  (0.45) |
| Inanimate - DOM | 770.04  (321.26) | 257.68  (108.09) | 512.36  (296.41) | 0.22  (0.42) | 0.26  (0.44) |
| Inanimate +DOM | 814.70  (304.94) | 256.60  (114.46) | 558.10  (283.41) | 0.29  (0.45) | 0.30  (0.46) |
| Region 5 |  |  |  |  |  |
| Animate + DOM | 580.38  (236.94) | 231.83  (90.95) | 348.55  (216.15) | 0.41  (0.49) | 0.20  (0.40) |
| Animate - DOM | 571.41  (238.64) | 248.50  (96.16) | 322.90  (213.94) | 0.33  (0.47) | 0.16  (0.36) |
| Inanimate - DOM | 611.63  (266.93) | 249.98  (92.75) | 361.65  (236.08) | 0.31  (0.47) | 0.16  (0.37) |
| Inanimate +DOM | 643.00  (239.44) | 241.07  (84.91) | 401.93  (225.88) | 0.32  (0.47) | 0.19  (0.39) |
| Region 6 |  |  |  |  |  |
| Animate + DOM | 619.56  (250.71) | 239.55  (88.40) | 380.01  (237.11) | 0.32  (0.47) | 0.27  (0.44) |
| Animate - DOM | 613.08  (260.21) | 236.76  (89.25) | 376.32  (243.08) | 0.28  (0.45) | 0.30  (0.46) |
| Inanimate - DOM | 613.95  (233.67) | 250.73  (97.82) | 363.22  (214.38) | 0.39  (0.49) | 0.18  (0.39) |
| Inanimate +DOM | 612.03  (250.73) | 243.13  (101.37) | 368.91  (233.59) | 0.34  (0.47) | 0.26  (0.44) |
|  |  |  |  |  |  |
|  |  |  |  |  |  |

** TT=Total Reading Times, SP= Second Pass Reading Times, FP= First Pass Reading Times, RI=Regressions In, RO=Regressions Out

**Table 9**: Reaction times and standard deviations for SVO sentences (L2)

|  | TT | | | SP | FP | | RI | | RO |
| --- | --- | --- | --- | --- | --- | --- | --- | --- | --- |
| Region 3 |  |  |  | | | | |  |  |
| Animate + DOM | 844.38  (319.57) | | | 244.31  (114.42) | 600.07  (299.59) | | 0.54  (0.50) | | 0.09  (0.30) |
| Animate - DOM | 822.36  (321.88) | | | 235.38  (110.83) | 586.98  (299.06) | | 0.55  (0.50) | | 0.10  (0.28) |
| Inanimate - DOM | 826.72  (302.56) | | | 243.30  (106.22) | 583.42  (280.51) | | 0.58  (0.50) | | 0.12  (0.32) |
| Inanimate +DOM | 865.12  (326.71) | | | 247.59  (114.15) | 617.53  (290.09) | | 0.58  (0.50) | | 0.08  (0.27) |
| Region 4 |  | | |  |  |  |  | |  |
| Animate + DOM | 814.64  (313.00) | | | 248.58  (102.37) | 566.06  (297.54) | | 0.31  (0.48) | | 0.21  (0.43) |
| Animate - DOM | 831.96  (315.16) | | | 244.30  (113.15) | 587.67  (296.97) | | 0.35  (0.47) | | 0.24  (0.41) |
| Inanimate - DOM | 829.77  (330.67) | | | 258.14  (114.53) | 571.63  (301.40) | | 0.32  (0.47) | | 0.25  (0.43) |
| Inanimate +DOM | 833.53  (309.66) | | | 259.85  (110.93) | 573.68  (295.84) | | 0.32  (0.47) | | 0.27  (0.45) |
| Region 5 |  | | |  |  |  |  | |  |
| Animate + DOM | 645.82  (276.38) | | | 236.46  (77.79) | 409.36  (259.38) | | 0.39  (0.48) | | 0.13  (0.34) |
| Animate - DOM | 629.84  (262.41) | | | 227.72  (102.79) | 402.11  (245.24) | | 0.37  (0.49) | | 0.13  (0.34) |
| Inanimate - DOM | 631.02  (258.34) | | | 231.37  (88.56) | 399.65  (244.02) | | 0.50  (0.50) | | 0.17  (0.38) |
| Inanimate +DOM | 647.58  (284.67) | | | 236.94  (94.25) | 410.65  (265.41) | | 0.43  (0.50) | | 0.12  (0.32) |
| Region 6 |  | | |  |  |  |  | |  |
| Animate + DOM | 670.65  (309.43) | | | 232.80  (83.34) | 437.85  (291.04) | | 0.35  (0.49) | | 0.27  (0.41) |
| Animate - DOM | 688.79  (275.20) | | | 227.90  (84.70) | 460.89  (269.62) | | 0.38  (0.48) | | 0.21  (0.45) |
| Inanimate - DOM | 595.81  (249.97) | | | 234.26  (89.31) | 361.55  (228.79) | | 0.42  (0.49) | | 0.21  (0.41) |
| Inanimate +DOM | 634.97  (258.24) | | | 229.43  (85.53) | 405.53  (245.78) | | 0.46  (0.50) | | 0.24  (0.43) |

** TT=Total Reading Times, SP= Second Pass Reading Times, FP= First Pass Reading Times, RI=Regressions In, RO=Regressions Out

**Table 10:** Results obtained in the MARKEDNESS*ANIMACY interaction in Region 4

| markedness | animacy | emmean | SE | DF | lower CL | upper CL |
| --- | --- | --- | --- | --- | --- | --- |
| [-DOM] | animate | 823.14 | 18.87 | 58.51 | 785.38 | 860.91 |
| [+DOM] | animate | 792.45 | 18.93 | 59.58 | 754.57 | 830.34 |
| [-DOM] | inanimate | 805.78 | 18.62 | 59.77 | 768.52 | 843.05 |
| [+DOM] | inanimate | 830.41 | 19.02 | 66.47 | 792.43 | 868.39 |

**Table 11:** Results obtained in the ANIMACY* GROUP interaction in Region 6

| animacy | group | emmean | SE | DF | lower CL | upper CL |
| --- | --- | --- | --- | --- | --- | --- |
| animate | HS | 605.03 | 103.27 | 24.64 | 556.1679 | 653.9010 |
| inanimate | HS | 599.30 | 107.94 | 24.89 | 549.9625 | 648.6484 |
| animate | L2 | 678.06 | 95.46 | 22.66 | 633.0720 | 723.0619 |
| inanimate | L2 | 615.06 | 94.99 | 22.63 | 570.1258 | 660.0108 |

**Table 12**: Results obtained in the MARKEDNESS*GROUP interaction in Region 5

| markedness | group | emmean | SE | DF | lower CL | upper CL |
| --- | --- | --- | --- | --- | --- | --- |
| [-DOM] | HS | 251.42 | 9.64 | 96.26 | 232.26 | 270.57 |
| [+DOM] | HS | 239.51 | 9.56 | 92.51 | 220.52 | 258.49 |
| [-DOM] | L2 | 224.86 | 8.22 | 88.20 | 208.51 | 241.20 |
| [+DOM] | L2 | 232.60 | 8.19 | 87.61 | 216.31 | 248.89 |

**Table 13**: Results obtained in the MARKEDNESS*ANIMACY interaction in Region 4

| markedness | animacy | emmean | SE | DF | lower CL | upper CL |
| --- | --- | --- | --- | --- | --- | --- |
| [-DOM] | animate | 569.90 | 18.41 | 52.38 | 532.95 | 606.85 |
| [+DOM] | animate | 535.28 | 18.47 | 53.23 | 498.22 | 572.34 |
| [-DOM] | inanimate | 546.16 | 18.15 | 54.28 | 509.77 | 582.55 |
| [+DOM] | inanimate | 570.31 | 18.49 | 59.76 | 533.31 | 607.31 |

**Table 14:** Results obtained in the ANIMACY* GROUP interaction in Region 6

| animacy | group | emmean | SE | DF | lower CL | upper CL |
| --- | --- | --- | --- | --- | --- | --- |
| animate | HS | 364.15 | 21.76 | 102.10 | 320.99 | 407.32 |
| inanimate | HS | 351.29 | 22.02 | 107.42 | 307.63 | 394.95 |
| animate | L2 | 451.76 | 19.96 | 93.13 | 412.11 | 491.40 |
| inanimate | L2 | 385.81 | 19.93 | 92.59 | 346.22 | 425.40 |

**Table 15**: Results obtained in the MARKEDNESS * GROUP interaction in Region 4

| markedness | animacy | emmean | SE | DF | lower CL | upper CL |
| --- | --- | --- | --- | --- | --- | --- |
| [-DOM] | animate | 0.33 | 0.02 | 46.24 | 0.27 | 0.39 |
| [+DOM] | animate | 0.28 | 0.02 | 46.98 | 0.22 | 0.34 |
| [-DOM] | inanimate | 0.27 | 0.02 | 48.32 | 0.22 | 0.33 |
| [+DOM] | inanimate | 0.30 | 0.021 | 53.02 | 0.24 | 0.36 |

**Table 16**: Results obtained in the ANIMACY * GROUP interaction in Region 5

| animacy | group | emmean | SE | DF | lower CL | upper CL |
| --- | --- | --- | --- | --- | --- | --- |
| animate | HS | 0.35 | 0.04 | 102.22 | 0.27 | 0.42 |
| inanimate | HS | 0.28 | 0.04 | 113.84 | 0.20 | 0.37 |
| animate | L2 | 0.39 | 0.03 | 85.48 | 0.32 | 0.45 |
| inanimate | L2 | 0.47 | 0.03 | 94.97 | 0.40 | 0.54 |

**Table 17**: Results obtained in the MARKEDNESS* ANIMACY*GROUP interaction in Region 4

| markedness | animacy | group | emmean | SE | DF | lower CL | upper CL |
| --- | --- | --- | --- | --- | --- | --- | --- |
| [-DOM] | animate | HS | 0.13 | 0.03 | 107.36 | 0.07 | 0.20 |
| [+DOM] | animate | HS | 0.08 | 0.03 | 106.05 | 0.02 | 0.15 |
| [-DOM] | inanimate | HS | 0.13 | 0.03 | 108.92 | 0.07 | 0.20 |
| [+DOM] | inanimate | HS | 0.16 | 0.03 | 108.90 | 0.10 | 0.23 |
| [-DOM] | animate | L2 | 0.13 | 0.03 | 126.63 | 0.06 | 0.20 |
| [+DOM] | animate | L2 | 0.10 | 0.03 | 131.78 | 0.04 | 0.17 |
| [-DOM] | inanimate | L2 | 0.22 | 0.03 | 115.50 | 0.16 | 0.29 |
| [+DOM] | inanimate | L2 | 0.16 | 0.03 | 125.49 | 0.09 | 0.22 |

**Table 18**: Results obtained in the MARKEDNESS* ANIMACY*GROUP interaction in Region 6

| markedness | animacy | group | emmean | SE | DF | lower CL | upper CL |
| --- | --- | --- | --- | --- | --- | --- | --- |
| [-DOM] | animate | HS | 0.30 | 0.05 | 69.87 | 0.20 | 0.41 |
| [+DOM] | animate | HS | 0.26 | 0.05 | 70.29 | 0.16 | 0.37 |
| [-DOM] | inanimate | HS | 0.18 | 0.05 | 82.18 | 0.08 | 0.29 |
| [+DOM] | inanimate | HS | 0.26 | 0.05 | 82.55 | 0.16 | 0.37 |
| [-DOM] | animate | L2 | 0.19 | 0.05 | 58.18 | 0.09 | 0.29 |
| [+DOM] | animate | L2 | 0.25 | 0.05 | 60.92 | 0.15 | 0.35 |
| [-DOM] | inanimate | L2 | 0.20 | 0.04 | 66.86 | 0.10 | 0.30 |
| [+DOM] | inanimate | L2 | 0.22 | 0.04 | 69.17 | 0.12 | 0.32 |

**Table 19**: Reaction times and standard deviations for VSO sentences (HS)

|  | TT | SP | FP | RI | RO |
| --- | --- | --- | --- | --- | --- |
| Region 3 |  |  |  |  |  |
| Animate + DOM | 809.35  (330.87) | 250.83  (115.89) | 558.52  (297.56) | 0.47  (0.50) | 0.12  (0.33) |
| Animate - DOM | 781.07  (314.11) | 245.95  (93.37) | 535.11  (296.56) | 0.52  (0.50) | 0.14  (0.31) |
| Inanimate - DOM | 773.14  (308.79) | 238.09  (105.53) | 535.05  (287.68) | 0.46  (0.50) | 0.09  (0.29) |
| Inanimate +DOM | 793.11  (306.17) | 238.73  (97.87) | 554.38  (295.94) | 0.52  (0.50) | 0.12  (0.31) |
| Region 4 |  |  |  |  |  |
| Animate + DOM | 806.14  (319.91) | 260.89  (113.93) | 545.25  (292.82) | 0.28  (0.45) | 0.22  (0.41) |
| Animate - DOM | 761.62  (299.61) | 252.53  (93.47) | 509.09  (290.03) | 0.30  (0.46) | 0.22  (0.42) |
| Inanimate - DOM | 809.79  (309.13) | 258.59  (100.59) | 551.20  (295.87) | 0.25  (0.43) | 0.23  (0.42) |
| Inanimate +DOM | 801.15  (297.51) | 267.32  (101.60) | 533.83  (275.05) | 0.26  (0.44) | 0.29  (0.46) |
| Region 5 |  |  |  |  |  |
| Animate + DOM | 620.39  (280.90) | 246.21  (102.27) | 374.18  (248.82) | 0.38  (0.49) | 0.23  (0.42) |
| Animate - DOM | 618.91  (256.32) | 235.01  (81.96) | 383.90  (248.08) | 0.33  (0.47) | 0.19  (0.40) |
| Inanimate - DOM | 594.01  (233.24) | 250.83  (112.97) | 343.18  (198.45) | 0.36  (0.48) | 0.15  (0.36) |
| Inanimate +DOM | 616.52  (273.00) | 244.55  (102.69) | 371.97  (243.69) | 0.31  (0.46) | 0.23  (0.42) |
| Region 6 |  |  |  |  |  |
| Animate + DOM | 619.49  (277.75) | 240.85  (93.56) | 378.65  (252.05) | 0.26  (0.44) | 0.29  (0.45) |
| Animate - DOM | 629.39  (249.51) | 243.59  (88.14) | 385.80  (226.08) | 0.28  (0.45) | 0.26  (0.44) |
| Inanimate - DOM | 588.15  (244.52) | 242.56  (86.82) | 345.60  (232.23) | 0.28  (0.45) | 0.27  (0.45) |
| Inanimate +DOM | 645.05  (258.15) | 248.79  (100.01) | 396.27  (238.56) | 0.35  (0.48) | 0.22  (0.42) |

** TT=Total Reading Times, SP= Second Pass Reading Times, FP= First Pass Reading Times, RI=Regressions In, RO=Regressions Out

**Table 20**: Reaction times and standard deviations for VSO sentences (L2)

|  | TT | SP | | FP | RI | RO |
| --- | --- | --- | --- | --- | --- | --- |
| Region 3 |  |  |  |  |  |  |
| Animate + DOM | 790.72  (316.64) | | 227.32  (101.28) | 563.41  (296.41) | 0.55  (0.50) | 0.10  (0.34) |
| Animate - DOM | 833.22  (301.63) | | 223.50  (102.40) | 609.72  (283.56) | 0.51  (0.50) | 0.14  (0.31) |
| Inanimate - DOM | 806.35  (319.40) | | 227.04  (101.69) | 579.30  (297.75) | 0.54  (0.50) | 0.15  (0.36) |
| Inanimate +DOM | 827.83  (307.95) | | 233.09  (108.20) | 594.74  (307.13) | 0.53  (0.50) | 0.10  (0.30) |
| Region 4 |  |  |  |  |  |  |
| Animate + DOM | 854.01  (296.32) | | 248.02  (111.57) | 605.99  (290.96) | 0.32  (0.47) | 0.29  (0.46) |
| Animate - DOM | 835.27  (309.30) | | 260.11  (102.60) | 575.16  (280.84) | 0.33  (0.47) | 0.30  (0.45) |
| Inanimate - DOM | 853.84  (325.13) | | 259.82  (108.33) | 594.02  (308.89) | 0.31  (0.46) | 0.32  (0.47) |
| Inanimate +DOM | 851.56  (326.15) | | 250.70  (99.70) | 600.87  (306.32) | 0.27  (0.44) | 0.23  (0.42) |
| Region 5 |  |  |  |  |  |  |
| Animate + DOM | 678.25  (250.51) | | 249.22  (92.69) | 429.04  (236.66) | 0.45  (0.48) | 0.15  (0.33) |
| Animate - DOM | 657.14  (281.70) | | 236.98  (96.71) | 420.16  (264.81) | 0.35  (0.50) | 0.12  (0.35) |
| Inanimate - DOM | 602.98  (250.04) | | 226.96  (84.24) | 376.02  (232.43) | 0.36  (0.48) | 0.15  (0.35) |
| Inanimate +DOM | 681.55  (286.11) | | 245.67  (112.03) | 435.88  (261.54) | 0.38  (0.49) | 0.12  (0.33) |
| Region 6 |  |  |  |  |  |  |
| Animate + DOM | 672.62  (288.17) | | 247.21  (94.34) | 425.41  (263.66) | 0.37  (0.48) | 0.24  (0.44) |
| Animate - DOM | 660.24  (305.36) | | 235.26  (109.53) | 424.98  (259.76) | 0.36  (0.48) | 0.25  (0.43) |
| Inanimate - DOM | 592.64  (248.01) | | 212.43  (65.94) | 380.20  (230.04) | 0.44  (0.50) | 0.19  (0.39) |
| Inanimate +DOM | 628.06  (291.64) | | 231.91  (88.60) | 396.15  (272.14) | 0.36  (0.48) | 0.21  (0.41) |

** TT=Total Reading Times, SP= Second Pass Reading Times, FP= First Pass Reading Times, RI=Regressions In, RO=Regressions Out

**Table 21:** Results obtained in the MARKEDNESS*GROUP interaction in Region 4

| markedness | group | emmean | SE | DF | lower CL | upper CL |
| --- | --- | --- | --- | --- | --- | --- |
| [-DOM] | HS | 260.24 | 9.48 | 93.53 | 241.40 | 279.08 |
| [+DOM] | HS | 268.20 | 9.46 | 92.92 | 249.40 | 287.01 |
| [-DOM] | L2 | 257.21 | 9.44 | 98.61 | 238.47 | 275.95 |
| [+DOM] | L2 | 244.62 | 9.38 | 96.37 | 226.00 | 263.24 |

**Table 22:** Results obtained in the ANIMACY*GROUP interaction in Region 5

| animacy | group | emmean | SE | DF | lower CL | upper CL |
| --- | --- | --- | --- | --- | --- | --- |
| animate | HS | 238.25 | 9.02 | 103.74 | 220.35 | 256.15 |
| inanimate | HS | 244.66 | 9.17 | 109.76 | 226.47 | 262.85 |
| animate | L2 | 238.57 | 8.18 | 97.23 | 222.32 | 254.82 |
| inanimate | L2 | 219.79 | 8.25 | 101.08 | 203.42 | 236.16 |

**Table 23**: Results obtained in the MARKEDNESS* ANIMACY*GROUP interaction in Region 3

| markedness | animacy | group | emmean | SE | DF | lower CL | upper CL |
| --- | --- | --- | --- | --- | --- | --- | --- |
| [-DOM] | animate | HS | 0.50 | 0.04 | 167.28 | 0.41 | 0.59 |
| [+DOM] | animate | HS | 0.44 | 0.04 | 143.32 | 0.35 | 0.53 |
| [-DOM] | inanimate | HS | 0.44 | 0.04 | 134.60 | 0.35 | 0.52 |
| [+DOM] | inanimate | HS | 0.50 | 0.04 | 136.97 | 0.41 | 0.58 |
| [-DOM] | animate | L2 | 0.53 | 0.04 | 147.98 | 0.44 | 0.61 |
| [+DOM] | animate | L2 | 0.57 | 0.04 | 143.11 | 0.48 | 0.65 |
| [-DOM] | inanimate | L2 | 0.56 | 0.04 | 138.44 | 0.47 | 0.64 |
| [+DOM] | inanimate | L2 | 0.54 | 0.04 | 142.13 | 0.46 | 0.63 |

**Table 24**: Results obtained in the MARKEDNESS* ANIMACY*GROUP interaction in Region 6

| markedness | animacy | group | emmean | SE | DF | lower CL | upper CL |
| --- | --- | --- | --- | --- | --- | --- | --- |
| [-DOM] | animate | HS | 0.28 | 0.05 | 92.71 | 0.13 | 0.34 |
| [+DOM] | animate | HS | 0.26 | 0.05 | 104.12 | 0.16 | 0.3 |
| [-DOM] | inanimate | HS | 0.32 | 0.05 | 103.02 | 0.21 | 0.43 |
| [+DOM] | inanimate | HS | 0.36 | 0.05 | 79.11 | 0.26 | 0.46 |
| [-DOM] | animate | L2 | 0.37 | 0.04 | 73.92 | 0.27 | 0.47 |
| [+DOM] | animate | L2 | 0.44 | 0.05 | 85.95 | 0.33 | 0.54 |
| [-DOM] | inanimate | L2 | 0.35 | 0.05 | 92.7 | 0.13 | 0.34 |
| [+DOM] | inanimate | L2 | 0.24 | 0.04 | 76.94 | 0.25 | 0.45 |

**Table 25**: Results obtained in the MARKEDNESS* ANIMACY*GROUP interaction in Region 3

| markedness | animacy | group | emmean | SE | DF | lower CL | upper CL |
| --- | --- | --- | --- | --- | --- | --- | --- |
| [-DOM] | animate | HS | 0.24 | 0.03 | 156.13 | 0.12 | 0.25 |
| [+DOM] | animate | HS | 0.20 | 0.03 | 131.99 | 0.14 | 0.27 |
| [-DOM] | inanimate | HS | 0.20 | 0.03 | 126.52 | 0.14 | 0.26 |
| [+DOM] | inanimate | HS | 0.26 | 0.03 | 121.85 | 0.20 | 0.32 |
| [-DOM] | animate | L2 | 0.24 | 0.03 | 155.88 | 0.18 | 0.31 |
| [+DOM] | animate | L2 | 0.12 | 0.03 | 149.32 | 0.06 | 0.19 |
| [-DOM] | inanimate | L2 | 0.24 | 0.03 | 134.24 | 0.17 | 0.30 |
| [+DOM] | inanimate | L2 | 0.19 | 0.03 | 138.09 | 0.12 | 0.25 |

**Table 26**: Results obtained in the MARKEDNESS* ANIMACY*GROUP interaction in Region 4

| markedness | animacy | group | emmean | SE | DF | lower CL | upper CL |
| --- | --- | --- | --- | --- | --- | --- | --- |
| [-DOM] | animate | HS | 0.24 | 0.03 | 133.11 | 0.18 | 0.25 |
| [+DOM] | animate | HS | 0.20 | 0.03 | 121.89 | 0.24 | 0.30 |
| [-DOM] | inanimate | HS | 0.26 | 0.02 | 125.75 | 0.21 | 0.31 |
| [+DOM] | inanimate | HS | 0.30 | 0.02 | 116.33 | 0.18 | 0.31 |
| [-DOM] | animate | L2 | 0.24 | 0.03 | 152.78 | 0.18 | 0.33 |
| [+DOM] | animate | L2 | 0.12 | 0.03 | 139.23 | 0.12 | 0.20 |
| [-DOM] | inanimate | L2 | 0.24 | 0.03 | 131.42 | 0.07 | 0.32 |
| [+DOM] | inanimate | L2 | 0.19 | 0.03 | 135.11 | 0.11 | 0.28 |
